# Understanding Suicidality Among Young People Living With and Affected by HIV: A Systematic Review

**DOI:** 10.1002/jia2.70193

**Published:** 2026-11-09

**Authors:** Kirsty M. Sievwright, Bai Xi Jasmine Chan, Mei Tan, Micaela Ciambrone, Virginia F. Byron, Maite Barrenechea, Justin Knox, Chloe A. Teasdale, Lorraine Sherr, Claude A. Mellins, Phillip Kreniske

**Affiliations:** ^1^ Department of Psychiatry Columbia University New York City New York USA; ^2^ New York State Psychiatric Institute New York City New York USA; ^3^ Department of Health Science Sacred Heart University Fairfield Connecticut USA; ^4^ Institute for Implementation Science in Population Health Graduate School of Public Health and Health Policy City University of New York New York City New York USA; ^5^ Wake Forest University Winston Salem North Carolina USA; ^6^ Division of Child and Adolescent Health Department of Pediatrics Columbia University Vagelos College of Physicians and Surgeons New York City New York USA; ^7^ Graduate School of Public Health and Health Policy City University of New York New York City New York USA; ^8^ Department of Sociomedical Sciences Mailman School of Public Health Columbia University New York City New York USA; ^9^ Department of Epidemiology and Biostatistics Graduate School of Public Health and Health Policy City University of New York (CUNY) New York City New York USA; ^10^ Department of Epidemiology, Mailman School of Public Health Columbia University New York City New York USA; ^11^ Institute of Global Health University College London London UK

**Keywords:** HIV, mental health, suicidality, suicide, systematic review, young people

## Abstract

**Introduction:**

Suicide is a leading cause of death among young people (ages 10–24), and suicidality—which encompasses suicidal ideation, planning and attempts—is a growing public health concern. Young people living with or affected by HIV may face heightened vulnerability to suicidality; however, evidence‐based mental health interventions for this group remain scarce. This systematic review aims to characterize the prevalence of suicidality and identify its determinants among young people with HIV‐related experiences to guide future research and intervention efforts.

**Methods:**

Using the Preferred Reporting Items for Systematic reviews and Meta‐Analyses guidelines, a systematic review of four online databases from 1 January 2008 to 14 August 2023 was completed. English‐language, peer‐reviewed studies of any design that satisfied the following criteria were included: (1) data primarily collected from 2008 onwards; (2) study sample with a mean/median age between 10 and 24 years; and (3) disaggregated findings reported on suicidality for those living with and/or affected by HIV.

**Results:**

From 3737 identified articles, 54 studies met the inclusion criteria. These studies spanned 19 countries, with 17 studies explicitly focused on suicidality and only six presenting data from young people affected by HIV. Reported prevalence varied widely: 23 studies documented suicidal ideation (range: 0%–36.4%), 13 studies reported on suicide attempts (range: 0.2%−30.4%) and 2 studies provided data on suicide deaths (0.17% and 2.8%). Among 12 studies that examined associated factors, depression was the factor most consistently linked to increased suicidality risk.

**Discussion:**

This review identified a wide prevalence range of suicidal ideation and suicide attempts among young people living with or affected by HIV. Yet, few of these studies explicitly focused on suicidality or assessed factors associated with suicidality. The consistent association between depression and suicidality suggests an urgent need for tailored mental health interventions.

**Conclusions:**

To address research gaps, future studies should move beyond cross‐sectional designs, examine protective factors, recruit participants outside of healthcare settings, and specifically include young people affected by HIV. In settings with limited mental health services, establishing clear and practical guidelines for the responsible conduct of research on suicidality is essential for advancing studies in this critical area.

**Abbreviations**
ADHD: attention‐deficit/hyperactivity disorder
ART: antiretroviral therapy
HIV: human immunodeficiency virus
MINI: Mini International Neuropsychiatric Interview
MINI‐KID: Mini International Neuropsychiatric Interview for Children and Adolescents
MMAT: Methods Appraisal Tool
NR: not reported
PHQ: Patient Health Questionnaire
PRISMA: Preferred Reporting Items for Systematic review and Meta‐Analyses Mixed
YPAHIV: young people affected by HIV
YPLHIV: young people living with HIV

## 1 Introduction

Since 2000, suicide mortality rates have steadily increased, and suicide is now a leading cause of death among young people (10−24 years) globally [1, 2, 3]. Suicidality, defined as the overall risk of suicide, encompasses suicidal ideation (thoughts or plans of self‐harm), suicide attempts (non‐fatal attempts to end one's life) and death by suicide. Young people living with or affected by chronic conditions such as HIV may be particularly vulnerable to suicidality, highlighting the need for targeted research and interventions in this population [4, 5].

The HIV epidemic notably impacts young people. Globally, an estimated 3.2 million youth (15–24 years) are living with HIV. Moreover, many more young people are affected through family members living with HIV; a reality reflected in the approximately 13.8 million children (0–17 years) orphaned by AIDS [6]. Compared to their peers, young people living with HIV (YPLHIV) and young people affected by HIV (YPAHIV) face intersecting social, structural and developmental challenges that uniquely heighten vulnerability to suicidality [7, 8, 9]. YPLHIV and YPAHIV personally manage or support family members with a stigmatized chronic illness, while navigating a stage of rapid physical, cognitive and psychological development [10, 11, 12, 13, 14, 15]. Many live in households faced with financial instability, changing family structures and persistent psychosocial stressors [16, 17, 18, 19]. This group experiences notable HIV‐related stigma, discrimination and bullying, all factors associated with increased risk of suicidality [20, 21, 22]. Material deprivation, increased household responsibilities and social marginalization may also limit access to basic needs and protective resources, intensifying psychological distress. Consistent with this, YPLHIV and YPAHIV exhibit higher prevalence of depression and anxiety than their peers, conditions that are well‐established predictors of suicidality and may exert syndemic effects [20, 23]. The burden of HIV is greatest in low‐resource settings where there is limited access to mental health and social support services that likely further exacerbate these risks [24].

A recent systematic review and meta‐analysis of 14 studies found that 24.38% and 13.05% of YPLHIV reported lifetime suicidal ideations and suicide attempts, respectively. However, this analysis did not consider suicidality among YPAHIV and limited its scope to the prevalence of suicidal ideations and attempts. Moreover, this review excluded qualitative studies and did not consider prevalence of death by suicide nor factors associated with suicidality [25]. Given rising suicidality among young people globally, there is an urgent need to understand: (1) the prevalence of suicidality across settings and (2) factors that contribute to or protect against suicidality among vulnerable populations, including YPLHIV and YPAHIV. This systematic review aims to synthesize the existing evidence on suicidality and its correlates among YPLHIV and YPAHIV to guide further research and intervention.

## 2 Methods

Using Preferred Reporting Items for Systematic review and Meta‐Analyses (PRISMA) guidelines [26], this review examined (1) the prevalence of suicidality among YLPHIV and YPAHIV globally as well as (2) the factors associated with suicidality among this population. The protocol for this systematic review was registered with PROSPERO (CRD42023455437) prior to initiation [27]. As this systematic review was a synthesis of existing peer‐reviewed journal articles and did not engage human subjects, no ethical approval or consent procedures were required.

### 2.1 Review Criterion

Studies were included if: (1) the mean or median age of the study sample was ≥10 and ≤24 years (to reflect real‐world study samples and avoid excluding studies predominantly focused on young people); (2) disaggregated data on suicidality (i.e. suicidal ideation, suicide attempts and/or death by suicide) was reported for those living with and/or affected by HIV; (3) the majority of data collection took place from 2008 and onwards, when HIV antiretroviral treatment was widely available globally [28]; and (4) the article was from a peer‐reviewed journal.

Studies were excluded if: (1) identified as a literature review, commentary, or grey literature and (2) the full‐text articles were not available in English.

### 2.2 Search Strategy and Screening

Four electronic databases were searched (PubMed, Cochrane Library, SCOPUS and Joanna Briggs Institute) from 1 January 2008 to 14 August 2023. Database‐specific search terms for young people, HIV and mental health were used, with searches conducted on two different dates: 14th August 2023 and 5th October 2023 (Supplementary Appendix A).

Using Covidence, six of the authors (KMS, BXJC, MT, MC, VFB and MB) participated in the screening process, first screening the titles and abstracts and then the full‐text articles. Two reviewers independently reviewed each article at each stage of screening. Conflicts were resolved by a third reviewer.

### 2.3 Data Extraction and Management

The following information was extracted from the studies included in this review:

- **Study identification**: Author(s), publication year;
- **Study context**: Study objective(s), study location(s), data collection period;
- **Study methods**: Design, analysis, procedures;
- **Study participants**: Inclusion criterion, sample size, demographic details; and
- **Findings regarding suicidality**: *n*(%) experienced, measures used to assess, correlates (disaggregated by each suicidality measure reported)

The data extraction form included all items from the Mixed Methods Appraisal Tool (MMAT) to appraise the quality of the quantitative, qualitative and mixed methods studies in this systematic review. In the MMAT, quality is ascertained through five questions tailored to each study type. Each question is scored “Yes” (1), “No” (0) or “Can't tell” (0.5) [29].

Seven of the authors (KMS, BXJC, MT, MC, VFB, MB and PK) extracted data. Data extraction for each included article was completed by two reviewers. Conflicts were resolved through mutual consensus between the two reviewers; when a consensus could not be reached, a third reviewer was consulted. In cases of missing or unclear information in the included studies, the lead author contacted the corresponding author(s) of the respective papers for clarity. An Excel spreadsheet was used to record data extracted from the included articles.

### 2.4 Data Synthesis

A narrative synthesis was conducted based on the exposure and coding categories [30]. The quantitative and qualitative data from the included studies were synthesized separately, and the resulting insights were integrated in the discussion. Where possible, throughout synthesis, differences in the prevalence data and factors associated with suicidality were assessed by suicidality outcome and with consideration of the study's focus on suicidality, population, setting and approach.

## 3 Results

Our search yielded 4386 studies (Figure 1). Among these, 649 duplicate studies were removed by Covidence or manually. Of the 3737 screened for title and abstract, 2777 were excluded. Of the 960 sought for retrieval, the full‐text paper could not be found in English for 17. During full‐text screening, 889 studies were excluded primarily due to: unmet age criterion; ineligible data collection periods; or a lack of data on suicidality. Ultimately, 54 studies were included in this review. Inter‐reviewer agreement was 87% for title and abstract screening, and 96% for full‐text screening.

**FIGURE 1:**
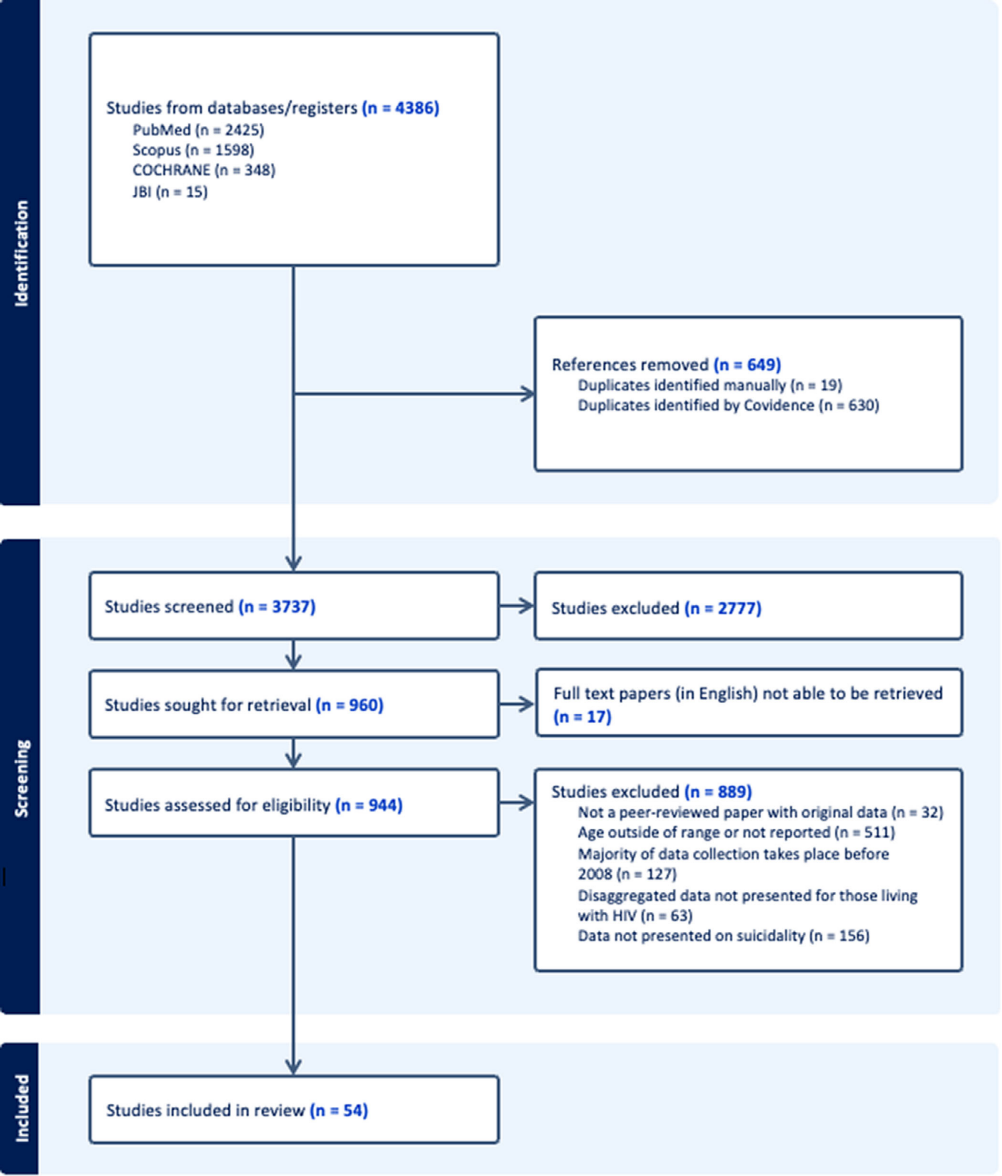
PRISMA flow chart of systematic review screening.

### 3.1 Description of Included Studies

The 54 included studies published between 2013 and 2023 are described in Tables 1, 2, 3. These studies spanned sub‐Saharan Africa (*n* = 38), the Americas and Caribbean (*n* = 11), Asia (*n* = 4) and Europe (*n* = 1), with 19 countries represented. Most (*n* = 29) focused on urban settings. Quantitative designs predominated (*n* = 35), with primarily cross‐sectional (*n* = 22) and cohort studies (*n* = 11). Most recruitment was clinic‐based (*n* = 42), and sampling was primarily purposive (*n* = 11) or unspecified (*n* = 29). Seventeen had a study aim focused on suicidality. There were five studies that included population eligibility criteria regarding mental health, of which one had a suicidality study.

**TABLE 1:** Quantitative study characteristics (*n* = 35).

Author, publication year	Location (urban, rural)	Study purpose (was the study focused on suicide)	Study design and data collection period	Sample living with or affected by HIV *N* (%)	Sample description (inclusion criteria, mean/median age, %female/%male) for those living with and/or affected by HIV	Recruitment, sampling methods for those living with and/or affected by HIV	MMAT (%)
**ASIA**							
Aurpibul 2023 [36]	Bangkok (two sites), Chiang Mai, Chiang Rai and Khon Kaen, Thailand Urban	Yes	Cross‐sectional November 2020−July 2021	Living with HIV: 355 (100%)	Young adults aged 18−25 years with perinatally acquired HIV and who had initiated ART. Median age: 21.7 Male: 163 (46%); Female: 192 (54%)	Identified from databases at five paediatric HIV clinics and recruited by the study team during regular clinic visits or telecommunication. Sampling approach not named.	80
Sohn 2023 [35]	Thailand, Vietnam, Malaysia Both rural and urban	No	Prospective cohort June 2016−February 2019	Living with HIV: 93 (100%)	Adolescents aged 16−24 years living with HIV, who received care within paediatric centres and transitioned to adult‐based centres in the past 12 months. Median age: 20 Male: 33 (40%); Female: 56 (60%)	Recruitment through four clinics. Convenience sampling.	90
Sudjaritruk 2021 [53]	Thailand Both rural and urban	Yes	Multicentre prospective cohort February 2018−April 2018	Living with HIV: 150 (50%)	Youth aged 15−25 years with perinatally or horizontally acquired HIV, aware of HIV status and received treatment. Mean age: 18.7 Male: 75 (50%); Female: 75 (50%)	Recruited from the clinics. Sampling approach NR.	90
**THE AMERICAS AND CARIBBEAN**							
Arnold 2023 [39]	Los Angeles, CA and New Orleans, LA, United States Urban	No	Longitudinal cohort May 2017−May 2020	Living with HIV: 170 (100%)	Young people aged 12−24 years with confirmed HIV diagnosis and ART treatment experience. Mean age: 21.9 Male: 120 (72.7%); Female: 45 (27.3%)	Recruited from homeless shelters, clinics, community‐based organizations, social media and dating applications. Sampling approach not named.	80
Gross 2016 [31]	United States Rural and urban	No	Cross‐sectional 2010−2012	Living with HIV: 387 (100%)	Young men aged 12−24 years living with HIV. Mean age: 21.32 Male: 387 (100%)	Recruited from 20 hospitals. Sampling approach not named.	100
Kipke 2020 [32]	Los Angeles, United States Urban	No	Longitudinal cohort May 2016−September 2017	Living with HIV: 51 (11.4%)	Male youth aged 16−24 years with either gay, bisexual or uncertain sexual orientation, reported a prior same‐sex sexual encounter, and self‐identified as African American/Black, Hispanic/Latino or multi‐racial/ethnic. Mean age: 22.6 Male: 51 (100%)	Recruited using venue‐based and social media strategies. Sampling approach not named.	90
Kreniske 2019 [46]	New York City, United States Urban	Yes	Longitudinal cohort 2003−2017	Living with HIV: *At enrolment*: 206 (61%); *follow‐up 5*: 151 (61%) Affected by HIV (perinatally exposed, but negative): *At enrolment*: 134 (39%); *follow‐up 5*: 97 (39%)	Youth aged 9−16 years exposed to HIV. Living with HIV: *At enrollment*: Mean age: 12.7 Male: 102 (50%); Female: 104 (50%) *At follow‐up 5*: Mean age: 22.79 Male: 68 (45%); Female: 83 (55%) Affected by HIV: *At enrolment*: Mean age: 12.4 Male: 66 (49%); Female: 68 (51%) *At follow‐up 5*: Mean age: 21.72 Male: 47 (48%); Female: 50 (52%)	Recruited through four HIV‐care medical centres. Sampling approach not named.	90
Kreniske 2021 [40]	New York City, United States Urban	Yes	Longitudinal cohort 2003−2019	Living with HIV: *At enrolment*: 206 (61%); *follow‐up 7*: 128 (62%) Affected by HIV (perinatally exposed, but negative): *At enrolment*: 133 (39%); *follow‐up 7*: 78 (38%)	Youth aged 9−16 years exposed to HIV. Living with HIV: *At enrolment*: Mean age: 12.70 Male: 102 (49.5%); Female: 104 (50.5%) Affected by HIV: *At enrolment*: Mean age: 12.38 Male: 65 (49%); Female: 68 (51%)	Recruited through four HIV‐care medical centres. Sampling approach not named.	100
Liotta 2023 [33]	New York City, United States Urban	Yes	Longitudinal cohort 2008−2018	Living with HIV: *At follow‐up 2*: 179 (63%); *follow‐up 4*: 146 (60%); *follow‐up 5*: 151 (60%) Affected by HIV (perinatally exposed, but negative): *At follow‐up 2*: 105 (37%); *follow‐up 4*: 96 (40%); *follow‐up 5*: 98 (40%)	Youth aged 9−16 years exposed to HIV. *At follow‐up 2*: Mean age: 17 *At follow‐up 4*: Mean age: 19 *At follow‐up 5*: Mean age: 22 Sex composition NR for youth living with HIV	Recruited through four HIV‐care medical centres. Sampling approach NR.	100
Mirani 2015 [47]	United States Both rural and urban	No	Longitudinal cohort April 2009−June 2014	Living with HIV: 1201 (100%)	Youth living with HIV. *For the first chart review*: Mean age: 17.4 *For the last chart review*: Mean age: 20.9 Male: 578 (48%); Female: 623 (52%)	Data derived from IMPAACT P1074 study. Recruitment and sampling approaches NR in present publication.	70
Orrigio 2021 [48]	Greater Kingston Metropolis, Jamaica Urban	No	Prospective cohort descriptive August 2003−February 2019	Living with HIV: 78 (100%)	Aged 24 years and younger with sexually acquired HIV. Median/mean age: NR Male: 16 (21%); Female: 62 (79%)	List of participants obtained from clinic databases. Sampling approach not named.	80
Walsh 2017 [34]	Southeast United States NR	No	Archival review of clinical database April 2014−December 2015	Living with HIV: 130 (100%)	Youth living with HIV. Mean age: 19.51 Male: 71 (54.6%); Female: 58 (44.6%)	Data retrieved from database at a paediatric infectious disease clinic. Sampling approach not named.	80
**SUB‐SAHARAN AFRICA**							
Adeyemo 2019 [61]	Lagos, Nigeria Urban	Yes	Cross‐sectional Data collection period NR^a^	Living with HIV: 201 (100%)	Adolescents aged 10−19 years with confirmed HIV diagnosis. Mean age: 13.88 Male: 124 (61.7%); Female: 77 (38.3%)	Recruited from two HIV out‐patient clinics. Consecutive sampling.	80
Ashaba 2021 [50]	Mbarara Town, Uganda Urban	Yes	Cross‐sectional November 2016−March 2017	Living with HIV: 224 (100%)	Adolescents aged 13−17 years living with HIV and who were aware of their HIV status. Mean age: 14.7 Male: 93 (42%); Female: 107 (58%)	Recruited from HIV clinic. Consecutive sampling.	80
Brittain 2019 [42]	Cape Town, South Africa Urban	No	Cross‐sectional July 2013−March 2015	Living with HIV: 506 (82.1%)	Adolescents aged 9−14 years, who received at least 6 months of ART and were aware of their HIV status. Median age: 11.9 Male: 258 (51%); Female: 248 (49%)	Recruited from ART services and youth centre. Sampling approach NR.	70
Brooks 2023 [111]	Gaborne, Botswana Urban	Yes	Cohort secondary data analysis January 2019−December 2019	Living with HIV: 1469 (100%)	Young people aged 12−25 years who did not screen for anxiety and depression within 12 months prior to study. Mean/median age: NR Male: 750 (51.1%); Female: 719 (48.9%)	Recruited during routine clinic visits. Sampling approach NR.	80
Casale 2019 [43]	Eastern Cape, South Africa Urban	Yes	Cross‐sectional 2014−2015	Living with HIV: 1053 (100%)	Adolescents aged 10−19 years living with HIV and who were initiated on treatment Mean age: 14 Male: 476 (45%); Female: 577 (55%)	Identified through medical databases from selected public healthcare facilities and traced back to their communities. Full community sampling.	100
Cluver 2013 [52]	Mpumalanga, Western Cape and KwaZulu‐Natal, South Africa Urban and rural	No	Cross‐sectional 2009−2012	Affected by HIV (AIDS‐orphaned): 811 (13.5%) Affected by HIV (AIDS‐sick parent): 1530 (25.5%)	Adolescents aged 10−17 years. AIDS‐orphaned: Mean age: 14.13 Male: 350 (43.2%); Female: 461 (46.8%) AIDS‐sick parent: Mean age: 13.55 Male: 604 (39.5%); Female: 926 (60.5%)	Recruited from selected health districts. Consecutive sampling approach.	100
Fawzi 2016 [112]	Southern Kayonza and Kirehe districts, Rwanda Rural	No	Cross‐sectional Data collection period NR	Living with HIV: 193 (100%)	Adolescents aged 10−17 years living with HIV‐positive and on ART. Mean/median age: NR Male: 97 (50%); Female: 96 (50%)	Recruited at health facilities in two target districts. Sampling approach NR.	80
Folayan 2022 [55]	Nigeria Both rural and urban	Yes	Cross‐sectional September 2020−October 2020	Living with HIV: 41 (3.3%)	Young people aged 13−19 years. Mean/median age: NR for youth living with HIV Sex composition for youth living with HIV NR	Recruitment took place through referrals (i.e. from peer educators, study staff or members of participant's network) and social media platforms (i.e. Facebook, Twitter, Instagram, WhatsApp). Respondent‐driven sampling, augmented with convenience sampling.	80
Gaitho 2018 [113]	Kenya Urban	No	Cross‐sectional August 2016−December 2016	Living with HIV: 270 (100%)	Adolescents aged 10−19 years. Mean age: 14.75 Male: 145 (53.7%); Female: 125 (47.3%)	Recruited at the clinic during appointment. Consecutive sampling.	80
Haas 2020 [49]	Johannesburg, South Africa Urban	No	Longitudinal cohort February 2018−January 2020	Living with HIV: 1203 (100%)	Adolescents aged 10−19 years who are receiving ART and had one or more mental health screenings. Mean age: 13 Male: 549 (50.5%); Female: 539 (49.5%)	Recruited from the clinic. Sampling approach not named.	70
Kemigisha 2019 [114]	Mbarara district, Uganda Urban	Yes	Cross‐sectional March 2017−May 2017	Living with HIV: 336 (100%)	Adolescents aged 10−19 years who are HIV care clinic patients. Median age: 13 Male: 127 (37.8%); Female: 209 (62.2%)	Recruited from urban and peripheral rural HIV/AIDS care medical centres. Sampling approach NR.	80
Kim 2014 [115]	Malawi Both rural and urban	No	Cross‐sectional January 2012−August 2012	Living with HIV: 562 (100%)	Adolescents aged 12−18 years living with HIV. Mean age: 14.5 Male: 247 (44%); Female: 315 (56%)	Recruited from a paediatric HIV clinic. Convenience sampling approach.	90
Namuli 2021 [60]	Mildmay, Uganda Urban	Yes	Cross‐sectional January 2014−March 2014	Living with HIV: 271 (100%)	Children and adolescents aged 6−18 years, living with HIV and living with the same primary caregiver for at least 1 year before study entry. Mean age: 11.6 Male: 120 (44%); Female: 151 (56%)	Recruited through the clinic by trained research assistants. Sampling approach not named.	80
Ndongo 2023 [38]	Yaounde, Cameroon Urban	No	Cross‐sectional December 2021−March 2022	Living with HIV: 302 (100%)	Adolescents aged 10−19 years living with perinatally acquired HIV and on ART. Median age: 15.2 Male: 143 (47%); Female: 159 (53%)	Recruited from a medical centre. Sampling approach NR.	80
Ng 2015 [41]	Kayonza, Kirehe districts, Rwanda NR	Yes	Matched case−control study March 2012−December 2012	Living with HIV: 218 (32%) Affected by HIV (children residing with HIV‐positive caregiver, or AIDS‐orphaned): 228 (33%)	Adolescents aged 10−17 years who lived in the included districts for over a month. Mean/median age: NR for those living with HIV or HIV‐affected Sex composition NR for those living with HIV or HIV‐affected	Electronic medical records used to identify villages of children living with HIV, and community health workers further recruited children living with HIV, affected by HIV and children who are not affected by HIV. Sampling approach NR.	90
Roberts 2022 [58]	Eastern Cape Province, South Africa Both rural and urban	No	Cross‐sectional February 2014−February 2017	Living with HIV: 510 (70.5%)	Young women aged 10−20 years and who had a pregnancy between 10 and 19 years old. Median age: NR for young women living with HIV Female: 510 (100%)	Participants living with HIV who initiated treatment within past 3 years were recruited from identified public health facilities. Sampling approach not named.	100
Rukundo 2020 [44]	Kampala and Masaka districts, Uganda Both rural and urban	Yes	Cross‐sectional 2015–2016	Living with HIV: 392 (100%)	Adolescents aged 12−17 years living with HIV. Mean/median age: NR Male: 183 (47%); Female: 209 (53%)	Recruited through five study clinics. Sampling approach NR.	80
Steventon Roberts 2022 [57]	Eastern Cape Province, South Africa Rural and peri‐urban	No	Cross‐sectional March 2018−July 2019	Living with HIV: 230 (24.1%)	Adolescent and young mothers (up to 24 years of age) Mean age: 19 Female: 230 (100%)	Recruited from health facilities, secondary schools, service provider referrals, maternity obstetric units, neighbouring adolescents of participants, referrals from adolescent mothers. Parallel sampling approach.	80
Steventon Roberts 2022 [56]	Eastern Cape Province, South Africa Rural and peri‐urban	No	Cross‐sectional March 2018−July 2019	Living with HIV: 272 (27.2%)	Adolescent mothers who had given birth between the ages of 10−19 years and had at least one living child. Median age: 19 Female: 272 (100%)	Recruited from health facilities, secondary schools, service provider referrals, maternity obstetric units, neighbouring adolescents of participants, referrals from adolescent mothers. Parallel sampling approach.	80
Sherr 2016 [116]	South Africa Both rural and urban	No	Secondary analysis of longitudinal data 2009−2012	Affected by HIV: 383 (20.7%)	Children aged 4−13 years affected by HIV and receiving community‐based organization (CBO) services. Mean age: 11.0 Male: 186 (48.6%); Female: 197 (51.4%)	Relevant data drawn from the Child Community Care study (CCC). Recruitment through CBOs. Two‐stage sampling approach: random sampling of CBOs, following by consecutive sampling of children.	100
Sherr 2018 [117]	Eastern Cape Province, South Africa Both rural and urban	No	Cross‐sectional Data collection period NR^a^	Living with HIV: 1024 (100%)	Youth aged 10−19 years who ever initiated ART in an urban or rural health district. Mean age: 13.76 Male: 471 (46%); Female: 553 (54%)	Identified and recruited from 53 district health facilities. Sampling approach NR.	100
Wonde 2019 [37]	Addis Ababa, Ethiopia Urban	Yes	Cross‐sectional May 2018−June 2018	Living with HIV: 413 (100%)	Youth living with HIV attending ART follow‐up visits in participating hospitals. Mean age: 20.9 Male: 152 (36.85); Female: 261 (63.2%)	Recruited from health facilities. Systematic random sampling.	100
Woollett 2017 [45]	Johannesburg, South Africa Urban	No	Cross‐sectional 2015–2016	Living with HIV: 343 (100%)	Adolescents aged 13−19 years living with HIV. Mean age: 16 Male: 165 (48%); Female: 178 (52%)	Recruited from five paediatric ARV healthcare facilities. Sampling approach not named.	80

Abbreviations: ART, antiretroviral therapy; HIV, human immunodeficiency virus; MMAT, Methods Appraisal Tool; NR, not reported.

^a^Data collection period was not reported in the published article; however, given the publication dates, it was assumed that the data collection period primarily took place in 2008 and after.

**TABLE 2:** Qualitative study characteristics (*n* = 13).

Author, publication year	Location (urban, rural)	Study purpose (was the study focused on suicide)	Study design and data collection period	Sample living with or affected by HIV *N* (%)	Sample description (inclusion criteria, mean/median age, %female/%male) for those living with and/or affected by HIV	Recruitment, sampling methods for those living with and/or affected by HIV	MMAT (%)
**ASIA**							
Asadi 2023 [65]	Tehran NR	No	Qualitative Data collection period NR^a^	Living with HIV: 17 (100%)	Young people aged 15−24 years living with HIV and Persian speaking. Mean age: 18.4 Male: 6 (35%); Female: 11 (65%)	Recruited from referrals to counselling centres. Targeted sampling.	70
**THE AMERICAS AND CARIBBEAN**							
Taquette 2017 [63]	Rio, Brazil Urban	No	Qualitative July 2010−December 2011	Living with HIV: 39 (100%)	Adolescents with HIV diagnosis between 10 and 19 years old and within the last 5 years. Mean/median age: NR Male: 16 (41%); Female: 23 (59%)	Recruited through medical facilities. Sampling approach not named.	70
**SUB‐SAHARAN AFRICA**							
Ashaba 2019 [51]	Mbarara District of Southwestern, Uganda Rural	No	Qualitative February 2016−March 2016	Living with HIV: Focus group discussion: 9 (53%); In‐depth interview: 5 (50%)	Adolescents aged 13−17 years living with HIV and receiving care at the HIV clinic. Mean/median age: NR Focus group discussion: Male: 4 (27%); Female: 11 (73%) In‐depth interview: Male: 2 (20%); Female: 8 (80%)	Recruited from clinics and local communities. Purposive sampling.	100
Jimu 2021 [70]	Chiredzi District, Zimbabwe Both rural and urban	No	Qualitative October 2019−December 2019	Living with HIV: 30 (100%)	Adolescents aged 13−19 years living with HIV, on ART and who had disclosed status to parents, guardians, and researchers. Mean/median age: NR Sex composition NR	Recruited through the hospital. Purposive sampling.	100
Kip 2022 [9]	Zomba, Malawi Urban	No	Qualitative April 2019−May 2019	Living with HIV: 80 (100%)	Adolescents aged 12−18 years living with HIV. Median age: 14 Male: 40 (50%); Female: 40 (50%)	Recruited by research staff from four clinic‐based adolescent ART‐sites. Purposive sampling.	90
Knizek 2017 [71]	Maska/Kampala, Uganda Both rural and urban	No	Qualitative July−November 2014	Living with HIV: 21 (100%)	Adolescents aged 12−17 years with positive HIV test at one of the participating clinics. Mean age: 14.6 Male: 9 (43%); Female: 12 (57%)	Recruited through the clinic. Random sampling.	70
Mutumba 2015 [72]	Kampala, Uganda Urban	No	Qualitative August 2011−November 2011	Living with HIV: 38 (100%)	Adolescents aged 13−19 years with perinatally acquired HIV and who were receiving HIV care. Mean age: 16.9 Male: 18 (47%); Female: 20 (53%)	Recruited at the clinic. Purposive sampling.	100
Nsibandze 2020 [68]	Manzani region, Eswatini Rural	No	Qualitative October 2017−April 2018	Living with HIV: 6 (100%)	Grandmothers of female adolescents aged 18−21 years, living with HIV and who knew their HIV status and were on ART^b^. Adolescent's mean age: 19.5 Female: 6 (100%)	Recruited from two clinics. Purposive sampling.	100
Nsibandze 2021 [69]	Manzini region at Mliba and Mafusteni areas, Eswatini Rural	No	Case study October 2017−April 2018	Living with HIV: 6 (100%)	Female youth aged 18−21 years living with HIV, on ART for at least 1 year prior to the commencement of the study and living full‐time with a grandmother. Mean age: 19.5 Female: 6 (100%)	Recruited from clinics. Purposive sampling.	80
Ouansafi 2021 [62]	Harare, Zimbabwe Urban	No	Qualitative Data collection period NR^a^	Living with HIV: 10 (100%)	Young adults aged 18−24 years living with HIV, on ART and who scored 9 or above on the Shona Symptoms Questionnaire‐14. Mean age: 21.1 Male: 3 (30%); Female: 7 (70%)	Recruited from primary care facilities. Purposive sampling.	100
Willis 2018 [73]	Harare, Zimbabwe Urban	No	Qualitative January 2015−June 2015	Living with HIV: 21 (100%)	Adolescents aged 15−19 years living with HIV and diagnosed depression. Mean/median age: NR Male: 11 (52%); Female: 10 (48%)	Recruited from clinical service delivery programme. Purposive sampling.	100
Yang 2018 [74]	Gaborne, Botswana Urban	No	Qualitative April 2014−February 2015	Living with HIV: 47(100%)	Adolescents aged 10−19 years living with HIV who were the most or least adherent patients in the cohort. For adherent group: Median age: 14 Male: 7 (47%); Female: 8 (53%) For non‐adherent group: Median age: 18 Male: 15 (47%); Female: 17 (53%)	Recruited from medical centre. Purposive sampling.	100
Yator 2022 [75]	Nairobi, Kenya Urban	No	Qualitative August 2018−July 2019	Living with HIV: 24 (100%)	Young mothers aged 15−24 years living with HIV and who were depressed postpartum. Mean/median age: NR Female: 24 (100%)	Recruited from two health centres. Purposive sampling.	100

Abbreviations: ART, antiretroviral therapy; HIV, human immunodeficiency virus; MMAT, Methods Appraisal Tool; NR, not reported.

^a^Data collection period was not reported in the published article; however, given the publication dates, it was assumed that the data collection period primarily took place in 2008 and after.

^b^Data collected from the grandmother's perspective.

**TABLE 3:** Mixed methods and medical case study characteristics (*n* = 6).

Author, publication year	Location (urban, rural)	Study purpose (was the study focused on suicide)	Study design and data collection period	Sample living with or affected by HIV *N* (%)	Sample description (inclusion criteria, mean/median age, %female/%male) for those living with and/or affected by HIV	Recruitment, sampling methods for those living with and/or affected by HIV	MMAT (%)
**EUROPE**							
Lee 2015 [66]	Munich, Germany Urban	Yes	Medical case study June−September 2014	Living with HIV: 1 (100%)	Aged 21 years with newly diagnosed HIV. Age: 21 Male: 1 (100%)	Participant identified from hospital case. Sampling approach not named.	NA
**THE AMERICAS AND CARIBBEAN**							
Kennard 2014 [64]	United States NR	No	Pilot assessment with case illustration January 2011−November 2013	Living with HIV: 8 (100%)	Youth aged 16−24 years with confirmed HIV and depression diagnoses, and engaged in care at the clinic. Mean age: 21.5 Male: 7 (87%); Female: 1 (13%)	Recruited through the clinic. Sampling approach not named.	70
**SUB‐SAHARAN AFRICA**							
Ashaba 2019 [59]	Mbarara, Uganda	No	Mixed methods February 2016−March 2017	Living with HIV: 224 (100%)	Youth aged 13−17 years old living with HIV and who were receiving care at the clinic. Mean age: 14.9 Male: 93 (42%); Female: 131 (58%)	Recruited from the HIV clinic and rural community sites. Purposive sampling.	70
Betancourt 2016 [67]	Kayonza and Kirehe districts, Rwanda Rural	No	Matched case−control with case studies March 2012−December 2012	Living with HIV and affected by HIV (those who are not living with HIV themselves but with a caregiver living with HIV): 19 (2.7%)	Adolescents aged 11−17 years affected by HIV. Mean/median age: NR for HIV affected youth Sex composition NR for HIV affected youth	Participants living with HIV were identified from medical databases, and then recruited into the study. Sampling approach not named.	50
Chhabra 2018 [76]	Nairobi, Kenya Urban	No	Mixed methods Data collection period NR^a^	Living with HIV: 32 (21.8%)	Orphan and vulnerable children aged 13−18 years. Mean/median age: NR for adolescents living with HIV Sex composition NR for adolescents living with HIV	Recruited by research staff at medical centre, community forums and from flyers. Sampling approach not named.	60
Zietz 2021 [54]	Nyanza region, Kenya Rural	Yes	Mixed methods 2016−2017	Living with HIV: 17 (0.42%)	Adolescents aged 15−19 years who had never tested HIV‐positive nor been tested in the past 6 months. Mean/median age: NR for adolescents living with HIV Sex composition NR for adolescents living with HIV	Recruited by staff from community awareness activities. Sampling approach not named.	60

Abbreviations: ART, antiretroviral therapy; HIV, human immunodeficiency virus; MMAT, Methods Appraisal Tool; NR, not reported.

^a^Data collection period was not reported in the published article; however, given the publication dates, it was assumed that the data collection period primarily took place in 2008 and after.

Most studies were comprised entirely of YPLHIV (*n* = 35). Three studies had samples with both YPLHIV and YPAHIV. In 16 studies, only a subset of participants were living with or affected by HIV. The mean and median ages of YPLHIV and YPAHIV ranged from 11.0 to 22.6 years; 15 studies did not report a mean or median age, but the age range fell within 10 and 24 years. Eight studies had single sex samples; four focused on pregnancy and motherhood and one on men who have sex with men. Four studies examined YPLHIV from the perspectives of their caregiver(s). Nineteen studies included sampling criteria regarding engagement in HIV care and treatment. Five studies included populations with perinatally acquired HIV.

The MMAT scoring is detailed in Supplementary Appendix B and summarized as a percentage in Tables 1, 2, 3. Average quality scores were 91.5% for qualitative, 70% for mixed‐methods and 80.9% for quantitative studies. None of the qualitative or quantitative studies received any “No” ratings. Seven qualitative studies received all “Yes” ratings compared to one quantitative study. All mixed‐methods studies received at least one “Can't tell” rating. Mixed‐methods studies often struggled with data integration and the traditional quality criteria set for each method, while quantitative scores were frequently lowered by limited reporting on response rates and the representativeness of the sample. The single medical case study in this review was not rated as it did not fit any of the MMAT study types.

### 3.2 Quantitative Findings

#### 3.2.1 Prevalence of Suicidality

##### 3.2.1.1 Suicidal Ideation

Twenty‐three studies spanning 13 countries reported prevalence data for suicidal ideation (Table 4). Assessments utilized 13 different tools, most commonly the Patient Health Questionnaire (*n* = 5) or Mini International Neuropsychiatric Interview (MINI, *n* = 4), over timeframes ranging from current to lifetime. Most focused exclusively on YPLHIV (*n* = 18). Reported mean or median ages for YPLHIV or YPAHIV ranged from 11 to 22.6 years (*n* = 20). Sixteen studies had a mean or median below 18 years and seven 18 years or older [31, 32, 33, 34, 35, 36, 37].

**TABLE 4:** Quantitative results—prevalence of suicidal ideation, by timeframe.

Author, year, location	Instrument	*N* (%)
**Current**		
Ndongo 2023 [38], Cameroon	CDI	110 (36.4%)
Namuli 2021 [60], Uganda	CDI	46 (17%)
Sohn 2023 [35], Thailand, Vietnam and Malaysia	PHQ9	11 (13.4%)
Adeyemo 2019 [61], Nigeria	MINI‐KID	30 (14.9%)
**Past week**		
Fawzi 2016 [112], Rwanda	CES‐DC	21 (11%)
**Past 2 weeks**		
Walsh 2017 [34], US	PHQ9	14 (10.8%)
Brooks 2023 [111], Botswana	PHQ9	224 (15%)
Gaitho 2018 [113], Kenya	PHQ9	12 (4.4%)
**Past month**		
Wonde 2019 [37], Ethiopia	CIDI	22 (5.7%)
Aurpibul 2023 [36], Thailand	PHQA	34 (9.6%)
Casale 2019 [43], South Africa	MINI‐KID	84 (8%)
Woollett 2017 [45], South Africa	MINI‐KID	60 (24%)
**Past 6 months**		
Kemigisha 2019 [114], Uganda	CES‐DC	26 (7.7%)
Ng 2015 [41], Rwanda	YSR Internalizing Subscale	Youth living with HIV: 46 (21.10%)
		Youth not living with HIV but who have a caregiver living with HIV: 51 (22.37%)
**Past year**		
Gross 2016 [31], US	Non‐standardized measure	51 (13.2%)
Chhabra 2018 [76], Kenya	GSHS	53 (20.3%)
Kipke 2020 [32], US	BSI	7 (14%)
Brittain 2019 [42], South Africa	Teen Talk	5 (1%)
Liotta 2023 [33], US	DISC‐IV‐YA	Youth living with HIV: Follow‐up period 2: 6 (3%) Follow‐up period 4: 5 (3%) Follow‐up period 5: 4 (3%)
		Youth not living with HIV but who have a caregiver living with HIV: Follow‐up period 2: 0 (0%) Follow‐up period 4: 4 (4%) Follow‐up period 5: 2 (2%)
**Lifetime**		
Adeyemo 2019 [61], Nigeria	MINI‐KID	67 (33.3%)
Haas 2020 [49], South Africa	Adolescent Innovations Project handbook	29 (2.7%)
Wonde 2019 [37], Ethiopia	CIDI	112 (27.1%)
**Not reported (NR)**		
Kim 2014 [115], Malawi	CDRS‐R	17 (3%)
	BDI‐II	40 (7.1%)
Sherr 2016 [116], South Africa	Non‐standardized question	14 (3.6%)
Sherr 2018 [117], South Africa	MINI	42 (4.1%)

Abbreviations: BDI‐II, Beck Depression Inventory II; BSI, Brief Symptom Inventory; CDI, Children's Depression Inventory; CDRS‐R, Children's Rating Scale, Revised; CES‐DC, Center for Epidemiologic Studies Depression Scale for Children; CIDI, Composite International Diagnostic Interview; DISC‐IV‐YA, Diagnostic Interview Schedule for Children Version 5; GSHS, Global School‐based Health Survey by World Health Organization; HIV, human immunodeficiency virus; MINI, Mini International Neuropsychiatric Interview; MINI‐KID, Mini International Neuropsychiatric Interview for Children and Adolescents; PHQ9, Patient Health Questionnaire Version 9; PHQA, Patient Health Questionnaire for Adolescents; YSR, youth self‐report.

Prevalence of suicidal ideation ranged from 0% to 36.4%, with a median of 8%–9.6%. The highest prevalence was 36.4% current ideation in Cameroon [38]. This cross‐sectional study focused on 10‐ to 19‐year‐old YPLHIV on antiretroviral therapy (ART) recruited from urban healthcare settings. It aimed to identify prevalence of and factors associated with depression [38]. The lowest prevalence was reported in a longitudinal study; prevalence of past‐year suicidal ideation was 0% and 2% at different follow‐up periods among perinatally HIV‐exposed but negative adolescents and young adults in the United States. Notably, this was the only study that presented prevalence data on suicidal ideation among YPAHIV [33]. The median prevalence of current suicidal ideation was 14.9%–17% compared to 27.1% over the lifetime. Reports of suicidal ideation were higher in sub‐Saharan Africa compared to other regions, with a median prevalence of 11%. Median suicidal ideation was higher in samples with mean or median age below 18 years (9.6% vs. 4.4%–7.1%).

##### 3.2.1.2 Suicide Attempts

Prevalence data on suicide attempts among YPLHIV and YPAHIV were presented in 13 studies across seven countries, with seven only including YPLHIV (Table 5). Suicide attempts were assessed over timeframes from the past month to lifetime; six reported lifetime suicide attempts. Eight different tools to measure suicide attempts were named across 11 studies; the MINI for Children and Adolescents (MINI‐KID) (*n* = 3) was most utilized. The mean or median age for YPLHIV and YPAHIV ranged from 11.9 to 22.6 years (*n* = 10). Five studies had samples with a mean or median of 18 years and above [32, 36, 37, 39, 40] and five below [41, 42, 43, 44, 45].

**TABLE 5:** Quantitative results—prevalence of attempted suicides, by timeframe.

Author, year, location	Instrument	*N* (%)
**Past month**		
Wonde 2019 [37], Ethiopia	CIDI	14 (3.4%)
Casale 2019 [43], South Africa	MINI‐KID	42 (4%) (Approximation)
Rukundo 2020 [44], Uganda	MINI‐KID	7 (1.8%)
Woollett 2017 [45], South Africa	MINI‐KID	17 (5%)
**Past 4 months**		
Arnold 2023 [39], US	Not reported	12 (23.5%)
**Past 6 months**		
Fawzi 2016 [112], Rwanda	Adapted CESD for children	23 (12%)
Ng 2015 [41], Rwanda	YSR Internalizing Subscale	Adolescents living with HIV: 46 (21.10%)
		Adolescents not living with HIV but who have a caregiver living with HIV: 49 (21.49%)
**Past year**		
Chhabra 2018 [76], Kenya	GSHS	62 (23.7%)
Kipke 2020 [32], US	BSI	1 (2%)
Brittain 2019 [42], South Africa	Teen Talk	1 (0.2%)
**Lifetime**		
Aurpibul 2023 [36], Thailand	Not reported	28 (7.9%)
Arnold 2023 [39], US	Not reported	51 (30.4%)
Wonde 2019 [37], Ethiopia	CIDI	70 (16.9%)
Kreniske 2019 [46], US	DISC‐IV‐YA	Youth living with HIV: 49 (24%)
		Youth perinatally HIV‐exposed but negative: 17 (13%)
Kreniske 2021 [40], US	DISC‐IV‐YA	Youth living with HIV: 55 (27%)
		Youth perinatally HIV‐exposed but negative: 21 (16%)
Rukundo 2020 [44], Uganda	MINI‐KID	11 (2.8%)

Abbreviations: BSI, Brief Symptom Inventory; CESD, Center for Epidemiologic Studies Depression Scale; CIDI, Composite International Diagnostic Interview; DISC‐IV‐YA, Diagnostic Interview Schedule for Children Version 5; GSHS, Global School‐based Health Survey by World Health Organization; HIV, human immunodeficiency virus; MINI‐KID, Mini International Neuropsychiatric Interview for Children and Adolescents; YSR, youth self‐report.

Prevalence of suicide attempts ranged from 0.2% to 30.4%, with a median of 13%. The lowest prevalence, 0.2%, was observed in a South African study measuring past‐year suicide attempts. Its purpose was not to assess suicidality, and only 82.1% of the sample were YPLHIV [42]. The highest prevalence of 30.4% was reported for lifetime attempts in a study that did not focus on suicidality but explored the relationship between trauma and depression predominantly among sexual‐ and gender‐minority 12‐ to 24‐year‐old YPLHIV in the United States [39]. Relatedly, reports of attempted suicides were highest in the United States, with a median prevalence of 23.5%.

The median prevalence of suicide attempts was 3.4%–4% over the past month and 16%–24% over the lifetime. While median prevalence was higher in studies focused on YPAHIV (16%) than YPLHIV (7.9%–12%), within the three studies that assessed both YPAHIV and YPLHIV, prevalence rates were higher or comparable for YPLHIV [40, 41, 46]. The median prevalence of suicide attempts was higher among samples with a mean or median age 18 years and above (14%−23.5% vs. 2.8%).

##### 3.2.1.3 Death by Suicide

Two studies included data on YPLHIV who died by suicide [44, 47]. In one, death by suicide was reported by caregivers among 2.8% (*n* = 11) of their youth, with seven of these suicides taking place in the past month. This cross‐sectional study had an explicit focus on suicide among 12‐ to 17‐year‐old YPLHIV in Uganda [44]. The other study was a longitudinal cohort analysis of YPLHIV who participated in the IMPAACT P1074 and PACTG 219C studies in the United States. This study reported that 0.17% (*n* = 2) of their sample died by suicide; this data was included as part of the mortality reporting for the analytical sample [47].

##### 3.2.1.4 Combined or Unspecified Suicidality

Data on the prevalence of or risk of suicidality (i.e. suicidal ideation, suicide attempts and/or death by suicide) were presented in 13 studies across six countries (Table 6). Most (*n* = 11) were set in sub‐Saharan Africa. Six studies focused exclusively on YPLHIV [43, 44, 48, 49, 50, 51], the remainder included YPAHIV [52] or a comparison group not living with HIV [53, 54, 55, 56, 57, 58]. The mean or median age for YPHIV and YPAHIV ranged from 13 to 19 years (*n* = 8). Six studies had a mean or median below 18 years [43, 44, 49, 50, 52, 59] and three 18 years and above [53, 56, 57].

**TABLE 6:** Quantitative results—prevalence of suicidality and suicide risk, by timeframe.

Author, year, location	Measure, instrument	*N* (%)
**Past 1–2 weeks**		
Zietz 2021 [54], Kenya	Suicide risk assessment, CESD	5 (29.4%)
**Past month**		
Casale 2019 [43], South Africa	Suicidal thoughts and behaviours (suicidal ideation and attempts), MINI‐KID	Mean score (0−5): 1.19
Roberts 2022 [58], South Africa	Suicidality symptoms (score ≥ 1), MINI KID	Mothers living with HIV: 9 (14.8%)
		Women living with HIV and not mothers: 20 (4.5%)
Rukundo 2020 [44], Uganda	Moderate to severe risk of suicidality (as assessed by youth's caregiver), MINI‐KID	42 (10.7%)
**Lifetime**		
Haas 2020, South Africa	Suicidality (suicidal ideation and attempts), Adolescent Innovations Project handbook	29 (2.7%)
Sudjaritruk 2021 [53], Thailand	Suicidality (suicidal ideation and attempts), PHQ9	17 (11.4%)
Orrigio 2021 [48], Jamaica	Suicidality (suicidal ideation and attempts), medical chart	18 (23.4%)
Ashaba 2021 [50], Uganda	Risk of suicidality, MINI‐KID	Low risk: 9 (4%) Medium risk: 13 (6%) High risk: 9 (4%)
Ashaba 2019 [59], Uganda		
Folayan 2022 [55], Nigeria	Suicidality (suicidal ideation and attempts), SBQ‐R	12 (29.3%)
**Not reported**		
Cluver 2013 [52], South Africa	Suicidality (not defined), MINI‐KID	AIDS orphaned youth: 66 (8.1%)
		Youth with an AIDS‐sick parent: 109 (7.1%)
Steventon Roberts 2022 [57], South Africa	Suicidality symptoms, MINI‐KID	Mean score (0−5): 0.31
		Any symptoms: 21 (9.1%)
Steventon Roberts 2022 [56], South Africa		Any symptoms: 23 (8.5%)

Abbreviations: CESD, Center for Epidemiologic Studies Depression Scale; HIV, human immunodeficiency virus; MINI‐KID, Mini International Neuropsychiatric Interview for Children and Adolescents; PHQ9, Patient Health Questionnaire Version 9; SBQ‐R, Suicide Behaviour Questionnaire Revised.

Five of these studies presented findings on suicidality that encompassed both suicidal ideation and suicide attempts among YPLHIV; four captured lifetime prevalence and one reported the mean MINI‐KID score of past‐month suicidality. Another study presented on suicidality without definition or timeframe among YPAHIV. Five instruments were used to assess suicidality across these six studies. Prevalence of such suicidality ranged from 2.7% to 29.3%, with a median between 8.1% and 11.4%. Seven studies reported assessments of suicidality risk among YPLHIV, for which mechanisms of reporting varied. Some studies reported prevalence of respondents experiencing: (1) any suicidality risk symptoms (*n* = 3); (2) specific categories of suicidality risk (*n* = 2); and (3) a certain level of suicidality risk (*n* = 2). The timeframe for documenting suicidality risk ranged from the past 1−2 weeks to throughout the lifetime. Nearly all studies measured suicidality risk using the MINI‐KID (*n* = 6).

#### 3.2.2 Correlates of Suicidality

Twelve studies representing seven countries presented regression results on factors associated with suicidal ideation, attempts and/or death by suicide among YPLHIV and/or YPAHIV using regression and hypothesis tests (Tables 7, 8). The majority took place in sub‐Saharan Africa (*n* = 9) and included a study objective on suicidality (*n* = 10) [36, 37, 40, 41, 43, 44, 46, 50, 60, 61]. Those that did not focus on suicidality included aims regarding mental health among young people [49, 62]. Most of the significant factors identified following regression analyses pertained to increased likelihood of suicidality (Table 7). The factors associated with suicidality in these studies were demographic (e.g. being female, older age and low socio‐economic status), clinical (e.g. living with HIV, HIV clinical stage and outcomes, mental health symptoms or diagnosis and physical abuse), psychosocial (e.g. death of family or friends, emotional abuse, self‐concept, disclosure, and HIV stigma and discrimination) or interpersonal (e.g. social support) in nature. Non‐significant factors are also presented for eight studies (Table 7).

**TABLE 7:** Factors quantitatively associated with increased vulnerability to suicidality.

Author, year, location	Suicidality outcome	Bivariate associations	Multivariate associations	Statistical methods
**Asia**				
Aurpibul 2023 [36], Thailand	Attempted suicide *Note: In this assessment, attempted suicide was assessed as a predictor of virologic non‐suppression*.	Virologic non‐suppression: OR: 3.02 (95% CI: 1.37−6.64) No non‐significant results reported	Virologic non‐suppression: aOR 2.66 (95% CI: 1.11–6.35)	Pearson's chi‐square test, Fisher's exact test, *Z*‐test, logistic regression
**The Americas and Caribbean**				
Kreniske 2019 [46], US	Attempted suicide	Living with HIV: OR: 2.35 (95% CI: 1.28−4.34) No non‐significant results reported	No multivariate regression reported	*T*‐tests, chi‐square tests, logistic regression
Kreniske 2021 [40], US	Attempted suicide	*Among YPLHIV and YPAHIV:* Living with HIV: OR 2.21 (95% CI: 1.18−4.12) Age: 1.18 (95% CI: 1.03−1.35) Social self‐concept: 1.75 (95% CI: 1.10−2.81) Depression: OR: 1.07 (95% CI: 1.01−1.14) DISC behaviour disorder: OR: 3.18 (95% CI: 1.22−8.29) No non‐significant results reported *Among YPLHIV only:* Depression: OR: 1.15 (95% CI: 1.06−1.25) DISC behaviour disorder: OR: 14.76 (95% CI: 1.37−158.90) No non‐significant results reported *Among YPAHIV only:* Depression: OR: 1.11 (95% CI: 1.03−1.21) No non‐significant results reported	No multivariate regression reported	*T*‐tests, chi‐square tests, Fisher's exact tests, backward stepwise logistic regression
**Sub‐Saharan Africa**				
Adeyemo 2019 [61], Nigeria	Suicidal ideation	*Current suicidal ideation:* Sex: χ2: 5.03 (*p*‐value: 0.025) Level of education: χ2: 1.68 (*p*‐value: 0.017) Family type: χ2: 5.36 (*p*‐value: 0.021) Oslo score: χ2: 6.95 (*p*‐value: 0.031) Decline in academic performance: χ2: 9.91 (*p*‐value: 0.005) Family member lost to HIV: χ2: 7.34 (*p*‐value: 0.007) Discrimination: χ2: 4.58 (*p*‐value: 0.044) Physical abuse: χ2: 3.87 (*p*‐value: 0.027) Emotional abuse: χ2: 9.83 (*p*‐value: 0.002) Loss of a family member to HIV: OR: 4.81 (95% CI: 1.68–13.76) Non‐significant factors: sex, educational level, family type, academic decline, physical abuse, emotional abuse, discrimination, total Oslo score *Lifetime suicidal ideation:* Reluctant to disclose status: χ2: 5.32 (*p*‐value: 0.021) Physical abuse: χ2: 6.78 (*p*‐value: 0.009) Emotional abuse: χ2: 5.38 (*p*‐value: 0.02) Non‐significant factors: disclosure, physical abuse, emotional abuse	No multivariate regression reported	Chi‐square tests, *t*‐tests, logistic regression
Ashaba 2021 [50], Uganda	Risk of suicidality	No bivariate regression reported	*Adjusted for covariates (age, orphanhood, type of school, bullying, HIV stigma, social support):* Childhood physical abuse: aRR: 3.17 (95% CI: 1.60−6.25) Childhood emotional abuse: aRR: 2.03 (95% CI: 1.05−3.92) Non‐significant factors: Childhood sexual abuse *Mutually adjusted model (covariates and the three forms of childhood abuse):* Childhood physical abuse: aRR 3.13 (95% CI 1.45–6.76) Non‐significant factors: childhood emotional abuse, childhood sexual abuse	Poisson regression, *E*‐value analysis
Casale 2019 [43], South Africa	Suicidality (suicidal thoughts and behaviour)	No bivariate regression reported	Depression: *B*: 0.16 (95% CI: 0.11−0.22) Stigma: *B*: 0.19 (95% CI: 0.13−0.24) Non‐significant factors: age, sex, urbanicity, SES, horizontal acquisition, caregiver biological parent, perceived social support, support group attendance	Unspecified regression approach
Haas 2020 [49], South Africa	Suicidality (suicidal ideation and attempts)	No bivariate regression reported	Age 10–12 years at screening: aOR: 0.10 (95% CI: 0.02−0.41) Age 13–15 years at screening: aOR: 0.37 (95% CI: 0.16−0.87) Experienced physical violence: aOR: 3.44 (95% CI: 1.55−7.65) Non‐significant factors: sex, conflict in household	Logistic regression
Namuli 2021 [60], Uganda	Suicidal ideation	Education: χ2: 6.15 (*p*‐value: 0.013) HIV disease stage I and II: χ2: 6.12 (*p*‐value: 0.013) Child has knowledge of HIV status: χ2: 6.24 (*p*‐value: 0.013) Exposure to family/friend's death: χ2: 22.14 (*p*‐value: <0.0001) Child sexual abuse or rape: χ2: 23.98 (*p*‐value: <0.0001) Non‐significant factors: age, sex, religion, orphanhood, CD4 count, physical abuse, change of residence, school‐related problems, parental separation/divorce, birth of a sibling, exposure to violence, other, HIV wasting syndrome Depression: MD: 10.2 (95% CI: 8.1−12.3) Anxiety symptoms: MD: 2.57 (95% CI: 1.6−3.6) Withdrawal symptoms: MD: 2.93 (95% CI: 1.7−4.1)	Exposure to family/friend's death: aPRR: 2.02 (95% CI: 1.01−4.03) Depression: aPRR: 1.08 (95% CI: 1.03−1.12) Anxiety symptoms: aPRR: 1.10 (95% CI: 1.01−1.20) Non‐significant factors: child age, child education, HIV disclosure status, sexual trauma, school‐related trauma, WHO HIV stage, CD4 count, HIV wasting syndrome, parental−child relationship, withdrawal symptoms, rule breaking behaviour, somatic symptoms, social problems, thought problems	Chi‐square tests, Poisson regression
		Somatic symptoms: MD: 2.07 (95% CI: 1.0−3.1) Thought problems: MD: 2.24 (95% CI: 1.3−3.2) Non‐significant factors: social problems, rule breaking behaviour, aggressive behaviour, other problems from CBCL, total CBCL		
Ng 2015 [41], Rwanda	Suicidal ideation	Being affected by HIV or living with HIV were not significantly associated with suicidal ideation No other specific bivariate analyses were reported for youth affected by or living with HIV	No multivariate regression reported for youth affected by or living with HIV	Logistic regression
	Suicidal behaviour	Being affected by HIV: OR: 1.89 (95% CI: 1.15−3.10) Living with HIV: OR: 1.85 (95% CI: 1.12−3.05) No other specific bivariate analyses were reported for youth affected by or living with HIV		
Rukundo 2020 [44], Uganda	Moderate to severe suicidality	HIV felt stigma: OR: 2.03 (95% CI: 1.03−3.01) Non‐significant factors: female, urban, age above 15, adolescent's education, caregiver education level, Christian, tribe, living with both parents, orphan, ever used a substance, sexual abuse, physical abuse, major depressive disorder, social anxiety disorder, PTSD, ADHD, oppositional defiant disorder, conduct disorder, caregiver psychological distress, lower CD4 count, poor child caregiver relationship, childhood trauma, negative life events, family with insufficient food	Low socio‐economic status—aOR: 2.27 (95% CI: 1.06−4.87) Major depressive disorder—aOR: 1.80 (95% CI: 0.48−2.10) HIV felt stigma—aOR: 2.10 (95% CI: 1.04–3.00) Non‐significant factors: female, urban, age above 15, adolescent's education, caregiver education level, Christian, tribe, living with both parents, orphan, ever used a substance, sexual abuse, physical abuse, social anxiety disorder, PTSD, oppositional defiant disorder, conduct disorder, lower CD4 count, poor child−caregiver relationship, childhood trauma, negative life events, family with insufficient food	Chi‐square tests, logistic regression
Sherr 2018 [117], South Africa	Suicidal ideation	Behavioural mode of acquisition: OR: 3.65 (95% CI: 1.96−6.82) No other bivariate regression results reported for the outcome of suicidal ideation.	Older age: aOR: 1.22 (95% CI: 1.05−1.41) Female: aOR: 2.43 (95% CI: 1.12−5.27) Non‐significant factors: poverty, maternal orphan, paternal orphan, mode of acquisition	Logistic regression, linear regression
Wonde 2019 [37], Ethiopia	Suicidal ideation	Female sex: aOR: 3.11 (95% CI: 1.90−5.20) History of a family death: OR: 2.1 (95% CI: 1.30−3.30) HIV clinical stage III: OR: 2.9 (95% CI: 1.40−5.70)	Female sex: aOR: 3.1 (95% CI: 1.60−6.00) History of a family death: aOR: 2.1 (95% CI: 1.20−3.90) HIV clinical stage III: aOR: 3.1 (95% CI: 1.30−7.40)	Logistic regression
		HIV clinical stage IV: OR: 8.4 (95% CI: 4.00−17.60) Having an opportunistic infection: OR: 1.7 (95% CI: 1.10−2.60) Living alone: OR: 4.5 (95% CI: 2.70−7.70) Disclosed HIV status: OR: 6.5 (95% CI: 3.50−11.90) Depression: OR: 10.7 (95% CI: 6.50−17.70) Poor social support: OR: 4.5 (95% CI: 2.30−9.10) Moderate social support: OR: 2.2 (95% CI: 1.20−4.20) HIV perceived stigma: OR: 5.3 (95% CI: 3.20−8.90) Non‐significant factors: HIV clinical stage II, history of family mental illness	HIV clinical stage IV: aOR: 4.8 (95% CI: 1.80−1.80) Depression: aOR: 7.1 (95% CI: 3.90−12.90) HIV perceived stigma: aOR: 4.3 (95% CI: 2.30−8.20) Non‐significant factors: HIV clinical stage II, opportunistic infection, living alone, non‐disclosure of HIV status, poor and moderate social support history of family mental illness	
	Suicide attempt	Female sex: aOR: 4.20 (95% CI: 2.10−8.60) HIV clinical stage III: OR: 3.2 (95% CI: 1.50−6.90) HIV clinical stage IV: OR: 6.2 (95% CI: 2.90−13.10) Having an opportunistic infection: OR: 3.6 (95% CI: 2.10−6.10) Living alone: OR: 3.1 (95% CI: 1.80−5.60) Disclosed HIV status: OR: 4.2 (95% CI: 2.20−7.80) Depression: OR: 8.8 (95% CI: 4.90−15.80) Poor social support: OR: 5.9 (95% CI: 2.70−13.30) HIV perceived stigma: OR: 2.7 (95% CI: 1.50−4.70) Non‐significant factors: HIV clinical stage II, moderate social support, history of family mental illness, history of a family death	Female sex: aOR: 4.1 (95% CI: 1.80−9.80) HIV clinical stage III: aOR: 3.1 (95% CI: 1.20−7.80) Having an opportunistic infection: aOR: 3.1 (95% CI: 1.60−6.00) Depression: aOR: 5.6 (95% CI: 2.80−11.10) Poor social support: aOR: 3.4 (95% CI: 1.20−9.40) Non‐significant factors: HIV clinical stage II and IV, living alone, non‐disclosure of HIV status, moderate social support, history of family mental illness, history of a family death	

Abbreviations: ADHD, attention‐deficit/hyperactivity disorder; aOR, adjusted odds ratio; aPRR, adjusted prevalence ratio; CBCL, Child Behavior Checklist; CI, confidence interval; DISC, Diagnostic Interview Schedule for Children; HIV, human immunodeficiency virus; MD, mean difference; OR, odds ratio; PTSD, post‐traumatic stress disorder; SES, socio‐economic status; YPAHIV, young people affected by HIV; YPLHIV, young people living with HIV.

Mental health symptoms and diagnosis were most frequently associated with increased suicidality among this population (*n* = 5), particularly depression, which was associated with increased suicidality in all five studies. Four of these studies presented multivariate results: Namuli et al. (2021) observed a relationship between depression and suicidal ideation as measured by the Children's Depression Inventory (CDI) (aPRR: 1.08 [1.03−1.12]); Casale et al. (2021) between depression and suicidal thoughts and behaviours as measured by the MINI‐KID (*B*: 0.16 [0.11−0.22]); Rukundo et al. (2020) between having a major depressive disorder and moderate to severe suicidality as measured by the MINI‐KID (aOR: 1.80 [0.48−2.10]); and Wonde et al. (2019) observed a relationship between both depression and suicidal ideation, and depression and suicide attempt as measured by the Composite International Diagnostic Interview (CIDI) (aOR: 7.1 [3.90−12.90] and aOR: 5.6 [2.80−11.10], respectively) [37, 43, 44, 60]. Finally, Kreniske et al. (2021) presented individual and combined bivariate results for YPLHIV and YPAHIV on the association between depression and attempted suicide as measured by the DISC‐IV‐YA, a full psychiatric interview (YPLHIV only: OR: 1.15 [1.06−1.25]; YPAHIV only: OR: 1.11 [1.03−1.21]; combined: OR: 1.07 [1.01−1.14]) [40].

The relationship between stigma and suicidality was assessed in three studies through a mix of bivariate and multivariate analyses. Wonde et al. (2019) identified an association between HIV perceived stigma and suicidal ideation (aOR: 4.3 [2.30−8.20]) and between HIV perceived stigma and suicide attempt (OR: 5.3 [3.20−8.90]); Casale et al. (2019) between stigma and suicidal thoughts and behaviour (*B*: 0.19 [0.13−0.24]); and Rukundo et al. (2020) between HIV felt stigma and moderate to severe suicidality (aOR: 2.10 [1.04−3.00]). In most cases, stigma had a greater effect than depression on suicidality [37, 43, 44].

Three of the 12 studies identified factors inversely associated with suicidality (Table 8) [40, 44, 60]. Namuli et al. (2021) found that 6‐ to 18‐year‐olds living with HIV in Uganda were less likely to report suicidal ideation if they had a stronger parent−child relationship (MD: 4.81 [−8.2 to −1.4]) and were experiencing HIV wasting syndrome, marked by substantial recent weight loss (aPRR: 0.39 [0.21−0.75]) [60]. Rukundo et al. (2020) observed that among 12‐ to 17‐year‐olds living with HIV in Uganda, those with attention‐deficit/hyperactivity disorder (ADHD) were less likely to have moderate to severe risk of suicidality (aOR: 0.41 [0.18–0.92]) [44]. Kreniske et al. (2021) identified that stronger family self‐concept (OR: 0.45 [0.25−0.81]) and religiosity (OR: 0.37 [0.17−0.78]) were associated with decreased likelihood of attempting suicide, respectively, among both YPLHIV and YPAHIV, and YPLHIV only in the United States [40].

**TABLE 8:** Factors quantitatively associated with decreased vulnerability to suicidality.

Author, year, location		Bivariate associations	Multivariate associations	
**The Americas and Caribbean**				
Kreniske 2021 [40], US	Suicide attempt among youth living with HIV	*Among YPLHIV and YPAHIV:* Family self‐concept: 0.45 (95% CI: 0.25−0.81) *Among YPLHIV only:* Religiosity: OR: 0.37 (95% CI: 0.17−0.78)	No multivariate regression reported	*T*‐tests, chi‐square tests, Fisher's exact tests, backward stepwise logistic regression
**Sub‐Saharan Africa**				
Namuli 2021 [60], Uganda	Suicidal ideation	Parent−child relationship: MD: −4.81 (95% CI: −8.2 to 1.4)	HIV wasting syndrome: aPRR: 0.39 (95% CI: 0.21−0.75)	
Rukundo 2020 [44], Uganda	Moderate to severe suicidality	No significant bivariate regression results reported	ADHD: aOR: 0.41 (95% CI: 0.18–0.92)	

Abbreviations: ADHD, attention‐deficit/hyperactivity disorder; aOR, adjusted odds ratio; aPRR, adjusted prevalence ratio; CI, confidence interval; HIV, human immunodeficiency virus; MD, mean difference; OR, odds ratio; YPAHIV, young people affected by HIV; YPLHIV, young people living with HIV.

### 3.3 Qualitative Findings

Seventeen studies presented qualitative results on suicidality among YPLHIV or YPAHIV [9, 51, 62, 63, 64, 65, 66, 67, 68, 69, 70, 71, 72, 73, 74, 75, 76]; however, data on suicidality were minimal. Only one study by Lee et al. (2015) was explicitly focused on suicidality; it offered a medical case presentation of a young man who has sex with men, newly diagnosed with HIV, who attempted suicide through overdosing on ART [66]. One other mixed‐methods study, though not explicitly focused on suicide, did highlight risk of harm among 11‐ to 17‐year‐olds in Rwanda. This study included case studies detailing suicidality among four respondents, three of whom were affected by HIV. These case studies emphasized the harms of HIV‐related stigma and how poor or abusive family relationships drive suicidal ideation and suicide attempts [67]. In the remaining qualitative studies, suicidality was mentioned illustratively rather than examined as a standalone theme. These themes typically pertained to understanding depression or broader mental health issues among this population [51, 62, 65, 74, 76], family support and environment [9, 51, 71], social connection or feelings of rejection [69, 70, 75] and negative reactions to learning HIV status [63, 68, 72].

The presence of family or other forms of social support was presented as a protective factor against suicidality, contributing to less self‐hatred [51, 64, 70]. Conversely, difficult family environments or a lack of social support and connection were potential drivers of suicidality [9, 69, 71, 76]. One study indicated that those who were female and 12 years or older when learning their HIV status were more likely to contemplate suicide [72].

Regarding prevalence, it was reported that seven respondents indicated suicidal ideation across two studies [62, 73]. Other studies did not specify the number of respondents reporting suicidal ideation. Across eight studies, based on the quotes provided, it was evident that at least 13 of the respondents (1−3 in each study) had contemplated suicide [51, 64, 67, 68, 69, 71, 74, 75]. Whereas, three studies noted that “many,” “most” or “more than half” of their sample of YPLHIV had contemplated suicide [63, 65, 70]. Few of these studies described the prevalence of suicide attempts. One indicated that “many” of the young people in their sample had “mentioned suicidal thoughts or even attempts” [65]. Whereas three others only offered information from one respondent who attempted suicide [66, 71, 74].

## 4 Discussion

This systematic review synthesizes data on suicidality among both YPLHIV and YPAHIV across 54 studies and 19 countries. There is a wide range of prevalence of suicidality across the included studies, which may be partly due to the use of different measures and definitions of suicidality across studies. Among the limited research on risk and protective factors, depression emerged as the most consistent correlate. This review highlighted ethical and methodological constraints that must be addressed to close research gaps and facilitate the development of targeted, evidence‐based intervention strategies.

Findings regarding the prevalence of suicidal ideation among YPLHIV and YPAHIV in the present review were comparable to those reported in the most recent available global syntheses of suicidality among young people in the general population. The range of past‐year suicidal ideation in the present review was 0%–36.4% compared to 1%–40% among 12‐ to 17‐year‐olds participating in the Global School‐based Student Health Survey that defined suicidal ideation as “ever seriously consider attempting suicide” and represented 82 countries [77]. The present review reported a median prevalence of 27.1% for lifetime suicidal ideation, compared to a weighted average of 20.4% among the global, general population of 6‐ to 21‐year‐olds in a meta‐analysis that defined active suicidal ideation as “thinking about killing oneself” [78]. Findings from the present review regarding the prevalence of lifetime suicide attempt differed from this literature. The present review reported a median between 16.9% and 24% compared to a weighted average of 7.0% in the aforementioned meta‐analysis, which did not directly define suicide attempt [78]. This difference in findings likely reflects the co‐occurrence and interaction of HIV‐related psychosocial and structural stressors, consistent with syndemic relationships that exacerbate psychological distress, and points to increased vulnerability for suicide attempts among YPLHIV and YPAHIV [4, 23].

The highest prevalence of suicidal ideation in this review was reported in sub‐Saharan Africa. This is a notable finding both because nearly 90% of YPLHIV reside in this region and the Global Burden of Disease study estimates a lower prevalence of mental disorders in this region [6, 79]. By contrast, the highest rates of suicide attempts in the present review were reported in the United States, where less than 0.1% of YPLHIV reside, but there is a higher prevalence of mental disorders among children and adolescents [6, 79]. Prior studies of the general population also show inconsistent geographic patterns: one reported higher suicide attempt rates in low‐ and middle‐income countries [80], while another found the highest suicide mortality in Central Europe [81]. In both studies, the suicidality outcomes were not explicitly defined. These geographic comparisons may reflect variations and limitations in data collection and surveillance systems resulting in underreporting of suicidality due to stigma and misclassification of deaths. Alternatively, these differences could be due to social and structural disparities that exist across countries and may influence suicidality among young people (e.g. through differences in accessibility of health services, stigma, and norms regarding mental health and HIV), underscoring the need for context‐specific research and interventions alongside global initiatives and goals.

Our review and other studies among the general population of young people observed that older age [77, 82], lower socio‐economic status [77], experienced abuse [21, 83] and poor mental health [83, 84] were associated with increased likelihood of suicidality. Poor mental health and specifically depression showed the most consistent significant association with suicidality of all factors in the present review. Notably, in the few studies that assessed HIV‐related stigma, its effect size on suicidality surpassed that of depression. This suggests that stigma is a primary, albeit under‐measured, driver. Future research should further elucidate these pathways by examining the mediating and moderating relationships between HIV‐related stigma, depression and suicidality in line with Casale et al. (2019) [43].

While other studies have found that being male was associated with an increased likelihood of suicidality among the general population of young people, in our assessment as well as that of Biswas et al. (2020), being female was associated with increased vulnerability [72, 77, 82, 83]. This is consistent with evidence demonstrating that adolescent girls and young women experience disproportionately high levels of HIV‐related stigma, placing them at increased risk for adverse mental health outcomes [85, 86]. Notably, while sexual‐ and gender‐minority youth consistently report an elevated risk of suicidality in the broader literature, sexual identity did not emerge as a significant correlate in the present review [87, 88, 89, 90]. This discrepancy may be attributed to a combination of developmental factors and methodological constraints within the primary studies. For instance, in many sub‐Saharan African contexts, as well as parts of the United States, querying youth regarding sexual identity remains taboo; consequently, this variable may have been underreported or omitted from primary studies altogether. Further, sexual identity for some may emerge later, so this factor may be missed during this developmental period.

In this review of studies, there was limited discussion of factors inversely associated with suicidality, indicating a need for additional research on protective factors [83]. Only three of the included studies observed such inverse and significant associations with five variables: religiosity [40], family self‐concept [40], a strong parent−child relationship [60], experiencing HIV wasting syndrome [60] and ADHD [44]. These findings did not fully converge with prior research among the general population, indicating that religiosity has mixed associations and ADHD typically has direct associations with suicidality [91, 92].

A handful of quantitative studies in the current review considered interpersonal level factors, such as family self‐concept [40] or the parent−child relationship [60]. Several qualitative studies also discussed the influence of social support and connection on suicidality among YPLHIV. Such factors are salient in the literature on suicidality in the general population of young people. Theorists and empirical studies have identified that having poor interpersonal relationships or no close friends is associated with suicide [77, 83, 84, 93]. These types of factors are highly applicable in the context of HIV. There is ample recent evidence on the importance of family and peer relationships in influencing internalized stigma and treatment adherence behaviours and outcomes among YPLHIV [94, 95, 96].

The scarcity of suicidality‐focused research likely reflects ethical concerns regarding participant distress and the lack of mental health referral infrastructure in low‐resource settings. Indeed, in this review, only one of the 17 studies with qualitative findings explicitly aimed to investigate suicidality [66], with the remaining 16 reporting suicidal behaviour only as an incidental by‐product of broader inquiries. This gap is noteworthy as qualitative modalities are uniquely suited to exploring sensitive health behaviours and are crucial to capturing the nuanced meanings of risk; without intentional qualitative inquiry, we risk overlooking how perceptions of suicidality differ within and across diverse groups, settings and contexts [97].

However, the probing nature of qualitative interviews carries the risk of inducing distress, and the tension between participant confidentiality and mandatory disclosure remains a significant barrier. While there is a growing body of evidence asserting that adolescents often demonstrate the capacity to provide informed consent [98, 99, 100, 101, 102, 103], navigating these protections in practice remains difficult. Confidentiality is upheld as a fundamental component of high‐quality care [104]; however, there is a critical need for research on how to apply these guidelines in diverse, low‐resource settings. Current researcher hesitation often stems from a lack of clear, field‐tested protocols for navigating HIV status, minority identity, and suicidality and operationalizing parental permission waivers. Future studies must explicitly evaluate the implementation of these ethical frameworks to ensure they provide actionable safeguards that protect youth without excluding the most vulnerable from the research process.

A greater diversity of study designs is essential to address the critical gaps identified in this review. The current literature is dominated by cross‐sectional designs (*n* = 22), providing only a snapshot of suicidality. Longitudinal assessments are needed to understand how vulnerability shifts across the HIV care cascade—from diagnosis and treatment initiation to the transition into adult care. Furthermore, as 42 studies recruited participants from healthcare settings, current evidence disproportionately reflects youth already connected to care. Future research must engage community‐based samples to capture the experiences of the most marginalized youth who remain outside traditional health systems. Finally, there is a notable gap in understanding the experiences of YAPHIV. Only six studies considered this group, leaving a significant void in our knowledge of how family HIV dynamics impact the suicidal contemplation and actions of affected youth.

Closing these gaps in the literature is critical to understanding the experience of and factors shaping suicidality among YPLHIV and YPAHIV. This understanding will support the development or adaptation of mental health interventions for this population. While there are some promising approaches for addressing adherence and mental health for adults living with HIV, to date, few evidence‐based interventions exist that are intended for YPLHIV or YPAHIV [105, 107]. Given widely varying resources in low‐ and middle‐income countries and high‐income countries, there may be a need for distinct approaches in tailoring mental health services for populations of YPLHIV and YPAHIV. This may include integrating HIV and mental healthcare, task‐shifting (having non‐specialists deliver mental healthcare), and utilizing technology to support mental health and HIV outcomes among YPLHIV and YPAHIV [108, 109, 110].

Several limitations of this review must be noted while interpreting the findings. First, while our search across four major databases was extensive, the exclusion of EMBASE and PsycINFO may have omitted some psychology‐specific literature. Second, the use of mean or median age for inclusion allowed samples to extend beyond the 10–24 range, potentially introducing developmental heterogeneity. Third, a formal meta‐analysis was not feasible due to the high degree of heterogeneity in suicidality measurement tools and the low number of studies reporting comparable quantitative correlates. Standardized suicidality metrics were rarely utilized across the 54 studies, and many reported prevalence without providing the necessary statistical data (such as standard errors or confidence intervals) required for a meaningful combined estimate. This review also has notable strengths. This is the first review to comprehensively synthesize the literature on suicidality among both YLPHIV and YAPHIV, offering a global overview of prevalence and correlates across diverse quantitative and qualitative data. Further, by targeting the 10–24 age range, the review captures a critical developmental window.

## 5 Conclusions

This systematic review underscores the urgent need for more research and targeted interventions to understand and address suicidality among YPLHIV and YPAHIV. Between 2008 and 2023, only 17 studies purposefully focused on suicidality in these groups, revealing both a scarcity of research and a wide variability in reported prevalence estimates. The wide variability in prevalence and the link between depression and HIV‐related stigma highlight the need for intersectional, evidence‐based mental health interventions (e.g. integrated HIV and mental healthcare, task‐shifting and digital health support). To advance the field, research on suicidality among youth living with and affected by HIV must transition from static, clinic‐based descriptions to longitudinal and community‐based designs that capture the lived experiences of youth across the entire HIV care cascade. Additionally, the well‐documented mental healthcare gap in low‐ and middle‐income countries, where the majority of YPLHIV and YPAHIV reside, poses significant challenges. In these contexts, implementing clear, practical and ethically sound guidelines for conducting research on sensitive topics like suicidality is essential to facilitate future studies and ultimately improve mental health outcomes for these vulnerable populations.

## Author Contributions

KMS, MC, JK, CAT, and PK determined the scope of the systematic review. KMS, MC and PK defined the search terms, and MC pulled the articles. KMS and PK prepared the PROSPERO protocol. KMS, BXJC, MT, MC, VFB and MB all contributed to the (a) title and abstract and (b) full‐text screenings. KMS, BXJC, MT, MC, VFB, MB and PK extracted the data. KMS led the development of the manuscript with writing support from BXJC and MT. All authors reviewed the manuscript and provided comments. BXJC, JK, LS, CAM and PK provided substantive feedback and revisions to the manuscript in conjunction with KMS.

## Funding

Kirsty M. Sievwright, Mei Tan, Justin Knox, and Claude A. Mellins received funding from the National Institute of Mental Health (NIMH) under award numbers T32MH019139 (Principal Investigator, Claude Mellins) and P30MH43520 (Principal Investigator, Robert Remien). Philip Kreniske and Bai Xi Jasmine Chan were partially supported by R01MH134695 (Principal Investigator, Philip Kreniske). Philip Kreniske and Chloe Teasdale were partially supported by R01MH140406 (Multiple Principal Investigators, Philip Kreniske and Proscovia Nabunya). Philip Kreniske was also partially supported by R01MH139432 (Multiple Principal Investigators, Philip Kreniske and Doron Amsalem). This systematic review was also carried out with the support of the Sacred Heart University EMERGE (Exploring Methods of Education & Research to Grow & Excel) Grant.

## Disclaimer

The views expressed are the authors’ and do not necessarily reflect the views and policies of the funding agencies.

## Conflicts of Interest

The authors report no conflicts of interest in this work.

## Supporting information




**Supporting File**: jia270193‐sup‐0001‐SuppMat.docx.

## Data Availability

Data sharing not applicable to this article as no datasets were generated or analysed during the current study.

## References

[1] Facts About Suicide | Suicide Prevention | CDC, https://www.cdc.gov/suicide/facts/index.html (accessed 15 August 2024).

[2] World Health Organization , Suicide Worldwide in 2019: Global Health Estimates (World Health Organization, 2019).

[3] Adolescent Health, https://www.who.int/multi‐media/details/causes‐of‐adolescent‐deaths (accessed 6 February 2025).

[4] A. J. Barnes , M. E. Eisenberg , and M. D. Resnick , “Suicide and Self‐Injury Among Children and Youth With Chronic Health Conditions,” Pediatrics 125 (2010): 889–895, 10.1542/peds.2009-1814. 20385631

[5] J. Iannucci and B. Nierenberg , “Suicide and Suicidality in Children and Adolescents With Chronic Illness: A Systematic Review,” Aggression and Violent Behavior 64 (2022): 101581, 10.1016/j.avb.2021.101581.

[6] AIDSinfo | HOMEPAGE, https://aidsinfo.unaids.org/ (accessed 20 January 2026).

[7] R. C. Vreeman , B. M. McCoy , and S. Lee , “Mental Health Challenges Among Adolescents Living With HIV,” Journal of the International AIDS Society 20 (2017): 21497, 10.7448/IAS.20.4.21497. PMC5577712 28530045

[8] C. A. Mellins and K. M. Malee , “Understanding the Mental Health of Youth Living With Perinatal HIV Infection: Lessons Learned and Current Challenges,” Journal of the International AIDS Society 16 (2013): 18593, 10.7448/IAS.16.1.18593. PMC3687078 23782478

[9] E. C. Kip , M. Udedi , K. Kulisewa , V. F. Go , and B. N. Gaynes , “Stigma and Mental Health Challenges Among Adolescents Living With HIV in Selected Adolescent‐Specific Antiretroviral Therapy Clinics in Zomba District, Malawi,” BMC Pediatrics 22 (2022): 253, 10.1186/S12887-022-03292-4. PMC9077887 35524228

[10] B. X. Tran , H. T. Phan , C. A. Latkin , et al., “Understanding Global HIV Stigma and Discrimination: Are Contextual Factors Sufficiently Studied? (GAPRESEARCH),” International Journal of Environmental Research and Public Health 16 (2019): 1899, 10.3390/IJERPH16111899. PMC6603743 31146379

[11] C. A. Laurenzi , S. Skeen , S. Gordon , et al., “Preventing Mental Health Conditions in Adolescents Living With HIV: An Urgent Need for Evidence,” Journal of the International AIDS Society 23 (2020): e25556, 10.1002/jia2.25556. PMC7459172 32869530

[12] S. Rueda , S. Mitra , and S. Chen , “Examining the Associations Between HIV‐Related Stigma and Health Outcomes in People Living With HIV/AIDS: A Series of Meta‐Analyses,” BMJ Open 6 (2016): e011453, 10.1136/BMJOPEN-2016-011453. PMC4947735 27412106

[13] A. L. Nguyen , E. Sundermann , A. A. Rubtsova , et al., “Emotional Health Outcomes Are Influenced by Sexual Minority Identity and HIV Serostatus,” AIDS Care 33 (2021): 1127–1132, 10.1080/09540121.2020.1785998. PMC7762728 32590909

[14] E. Kimera , S. Vindevogel , D. Reynaert , et al., “Experiences and Effects of HIV‐Related Stigma Among Youth Living With HIV/AIDS in Western Uganda: A Photovoice Study,” PLoS ONE 15 (2020): e0232359, 10.1371/JOURNAL.PONE.0232359. PMC7182188 32330206

[15] A. Kiwanuka , E. Walakira , L. Andersson , and D. K. Mafigiri , “Barriers of HIV Treatment‐Seeking Behavior Among Youths Living With HIV in Uganda: A Qualitative Study,” HIV & AIDS Review 21 (2022): 43–49, 10.5114/hivar.2022.112711.

[16] S. Ryu and L. Fan , “The Relationship Between Financial Worries and Psychological Distress Among U.S. Adults,” Journal of Family and Economic Issues 44 (2023): 16–33, 10.1007/s10834-022-09820-9. PMC8806009 35125855

[17] L. V. Ghazal , K. Wentzell , and J. E. Blanchette , “Mobilizing Research to Understand the Impact of Financial Toxicity Among Adolescents and Young Adults Diagnosed With Chronic Diseases,” Journal of Adolescent Health 73 (2023): 403–405, 10.1016/j.jadohealth.2023.05.020. PMC10697307 37389528

[18] P. Cavazos‐Rehg , C. Xu , E. Kasson , W. Byansi , O. S. Bahar , and F. M. Ssewamala , “Social and Economic Equity and Family Cohesion as Potential Protective Factors From Depression Among Adolescents Living With HIV in Uganda,” AIDS and Behavior 24 (2020): 2546–2554, 10.1007/s10461-020-02812-6. PMC7725408 32095914

[19] S. Z. Shahram , M. L. Smith , S. Ben‐David , M. Feddersen , T. E. Kemp , and K. Plamondon , “Promoting “Zest for Life”: A Systematic Literature Review of Resiliency Factors to Prevent Youth Suicide,” Journal of Research on Adolescence 31 (2021): 4–24, 10.1111/jora.12588. PMC7986824 33665921

[20] S. Ashaba , C. Cooper‐Vince , S. Maling , G. Z. Rukundo , D. Akena , and A. C. Tsai , “Internalized HIV Stigma, Bullying, Major Depressive Disorder, and High‐Risk Suicidality Among HIV‐Positive Adolescents in Rural Uganda,” Global Mental Health 5 (2018): e22, 10.1017/gmh.2018.15. PMC6036650 29997894

[21] M. F. Faidas , B. N. Gaynes , S. M. Mphonda , et al., “‘You Feel Like It's Better to Just Die’: Death‐Centric Stereotypes and Stigma Contribute to Suicide Risk for Adolescents Living With HIV in Malawi,” PLOS Global Public Health 5 (2025): e0005655, 10.1371/journal.pgph.0005655. PMC12747334 41460867

[22] P. Kreniske , C. Morrison , B. H. Spencer , et al., “HIV and Suicide Risk Across Adolescence and Young Adulthood: An Examination of Socio‐Demographic, Contextual and Psychosocial Risk Factors for Attempted Suicide in a Longitudinal Cohort of Ageing Adolescents Affected by HIV Living in the New York City Area,” Journal of the International AIDS Society 25 (2022): e25984, 10.1002/jia2.25984. PMC9522633 36176026

[23] W. Wang , C. Xiao , X. Yao , Y. Yang , H. Yan , and S. Li , “Psychosocial Health and Suicidal Ideation Among People Living With HIV/AIDS: A Cross‐Sectional Study in Nanjing, China,” PLoS ONE 13 (2018): e0192940, 10.1371/journal.pone.0192940. PMC5823403 29470532

[24] E. Brohan , N. Chowdhary , T. Dua , et al., “The WHO Mental Health Gap Action Programme for Mental, Neurological, and Substance Use Conditions: The New and Updated Guideline Recommendations,” Lancet Psychiatry 11 (2024): 155–158, 10.1016/S2215-0366(23)00370-X. 37980915

[25] L. Tsegay and G. Ayano , “The Prevalence of Suicidal Ideation and Attempt Among Young People With HIV/AIDS: A Systematic Review and Meta‐Analysis,” Psychiatric Quarterly 91 (2020): 1291–1304, 10.1007/s11126-020-09851-1. 32960412

[26] D. Moher , L. Shamseer , M. Clarke , et al., “Preferred Reporting Items for Systematic Review and Meta‐Analysis Protocols (PRISMA‐P) 2015 Statement,” Systematic Reviews 4 (2015): 148–160, 10.1186/2046-4053-4-1. PMC4320440 25554246

[27] J. H. Schiavo , “PROSPERO: An International Register of Systematic Review Protocols,” Medical Reference Services Quarterly 38 (2019): 171–180, 10.1080/02763869.2019.1588072. 31173570

[28] M. R. Reich and P. Bery , “Expanding Global Access to ARVs. The Challenges of Prices and Patents,” The AIDS Pandemic: Impact on Science and Society (2004): 324–350.

[29] Q. N. Hong , S. Fàbregues , G. Bartlett , et al., “The Mixed Methods Appraisal Tool (MMAT) Version 2018 for Information Professionals and Researchers,” Education for Information 34 (2018): 285–291, 10.3233/EFI-180221.

[30] K. Lisy and K. Porritt , “Narrative Synthesis: Considerations and Challenges,” JBI Evidence Implementation 14 (2016): 201.

[31] I. M. Gross , S. Hosek , M. H. Richards , and M. I. Fernandez , “Predictors and Profiles of Antiretroviral Therapy Adherence Among African American Adolescents and Young Adult Males Living With HIV,” AIDS Patient Care and STDs 30 (2016): 324–338, 10.1089/apc.2015.0351. PMC4948258 27410496

[32] M. D. Kipke , K. Kubicek , I. C. Akinyemi , et al., “The Healthy Young Men's Cohort: Health, Stress, and Risk Profile of Black and Latino Young Men Who Have Sex With Men (YMSM),” Journal of Urban Health 97 (2020): 653–667, 10.1007/s11524-019-00398-6. PMC7560671 32864727

[33] L. Liotta , L. Kluisza , N. Nguyen , et al., “Hope for the Future Protects Against Suicidal Ideation Among Adolescents and Young Adults Affected by Perinatal HIV,” AIDS Care 35 (2023): 1948–1954, 10.1080/09540121.2023.2184764. PMC10491736 36892951

[34] A. S. J. Walsh , K. L. Wesley , S. Y. Tan , et al., “Screening for Depression Among Youth With HIV in an Integrated Care Setting,” AIDS Care 29 (2017): 851–857, 10.1080/09540121.2017.1281878. 28278567

[35] A. H. Sohn , T. Singtoroj , K. Chokephaibulkit , et al., “Long‐Term Post‐Transition Outcomes of Adolescents and Young Adults Living With Perinatally and Non‐Perinatally Acquired HIV in Southeast Asia,” Journal of Adolescent Health 72 (2023): 471–479, 10.1016/j.jadohealth.2022.10.021. PMC11772534 36535867

[36] L. Aurpibul , P. Kosalaraksa , S. Kawichai , et al., “Alcohol Use, Suicidality and Virologic Non‐Suppression Among Young Adults With Perinatally Acquired HIV in Thailand: A Cross‐Sectional Study,” Journal of the International AIDS Society 26 (2023): e26064, 10.1002/jia2.26064. PMC9925945 36785872

[37] M. Wonde , H. Mulat , A. Birhanu , et al., “The Magnitude of Suicidal Ideation, Attempts and Associated Factors of HIV Positive Youth Attending ART Follow Ups at St. Paul's Hospital Millennium Medical College and St. Peter's Specialized Hospital, Addis Ababa, Ethiopia, 2018,” PLoS ONE 14 (2019): e0224371, 10.1371/journal.pone.0224371. PMC6830816 31689299

[38] F. A. Ndongo , R. Kana , M. T. Nono , et al., “Mental Health Troubles Among Cameroonian Adolescents Perinatally Infected With Human Immunodeficiency Virus,” Revue d'Épidémiologie et de Santé Publique 71 (2023): 101422, 10.1016/j.respe.2022.101422. 36706703

[39] E. M. Arnold , M. M. Yalch , J. Christodoulou , D. A. Murphy , D. Swendeman , and M. J. Rotheram‐Borus , “Rumination Influences the Relationship Between Trauma and Depression Over Time Among Youth Living With HIV,” Journal of Affective Disorders 322 (2023): 9–14, 10.1016/j.jad.2022.11.010. PMC9793436 36370915

[40] P. Kreniske , C. A. Mellins , C. Dolezal , et al., “Predictors of Attempted Suicide Among Youth Living With Perinatal HIV Infection and Perinatal HIV‐Exposed Uninfected Counterparts,” Journal of Acquired Immune Deficiency Syndromes 88 (2021): 348–355, 10.1097/QAI.0000000000002784. PMC8693508 34406984

[41] L. C. Ng , C. M. Kirk , F. Kanyanganzi , et al., “Risk and Protective Factors for Suicidal Ideation and Behaviour in Rwandan Children,” British Journal of Psychiatry 207 (2015): 262–268, 10.1192/bjp.bp.114.154591. PMC4555444 26045350

[42] K. Brittain , L. Myer , N. Phillips , et al., “Behavioural Health Risks During Early Adolescence Among Perinatally HIV‐Infected South African Adolescents and Same‐Age, HIV‐Uninfected Peers,” AIDS Care 31 (2019): 131–140, 10.1080/09540121.2018.1533233. PMC6299266 30340446

[43] M. Casale , M. Boyes , M. Pantelic , E. Toska , and L. Cluver , “Suicidal Thoughts and Behaviour Among South African Adolescents Living With HIV: Can Social Support Buffer the Impact of Stigma?,” Journal of Affective Disorders 245 (2019): 82–90, 10.1016/j.jad.2018.10.102. 30368074

[44] G. Z. Rukundo , R. S. Mpango , W. Ssembajjwe , K. D. Gadow , V. Patel , and E. Kinyanda , “Prevalence and Risk Factors for Youth Suicidality Among Perinatally Infected Youths Living With HIV/AIDS in Uganda: The CHAKA Study,” Child and Adolescent Psychiatry and Mental Health 14 (2020): 41, 10.1186/s13034-020-00348-0. PMC7585678 33110444

[45] N. Woollett , L. Cluver , M. Bandeira , and H. Brahmbhatt , “Identifying Risks for Mental Health Problems in HIV Positive Adolescents Accessing HIV Treatment in Johannesburg,” Journal of Child & Adolescent Mental Health 29 (2017): 11–26, 10.2989/17280583.2017.1283320. 28287023

[46] P. Kreniske , C. A. Mellins , C. Dolezal , et al., “Sounding the Alarm: Perinatally HIV‐Infected Youth More Likely to Attempt Suicide Than Their Uninfected Cohort Peers,” Journal of Adolescent Health 65 (2019): 702–705, 10.1016/j.jadohealth.2019.06.006. PMC6814538 31481285

[47] G. Mirani , P. L. Williams , M. Chernoff , et al., “Changing Trends in Complications and Mortality Rates Among US Youth and Young Adults With HIV Infection in the Era of Combination Antiretroviral Therapy,” Clinical Infectious Diseases 61 (2015): 1850–1861, 10.1093/cid/civ687. PMC4657532 26270680

[48] K. Orrigio , R. B. Pierre , D. Gordon‐Harrison , K. Lewis‐O'connor , G. Gordon‐Strachan , and C. D. C. Christie , “Sexual Abuse and Sexually‐Transmitted HIV/AIDS in Jamaican Children and Adolescents Aged 6−19 Years,” Journal of Infection in Developing Countries 15 (2021): 989–996, 10.3855/jidc.12156. 34343124

[49] A. D. Haas , K.‐G. Technau , S. Pahad , et al., “Mental Health, Substance Use and Viral Suppression in Adolescents Receiving ART at a Paediatric HIV Clinic in South Africa,” Journal of the International AIDS Society 23 (2020): e25644, 10.1002/jia2.25644. PMC7720277 33283916

[50] S. Ashaba , C. E. Cooper‐Vince , S. Maling , et al., “Childhood Trauma, Major Depressive Disorder, Suicidality, and the Modifying Role of Social Support Among Adolescents Living With HIV in Rural Uganda,” Journal of Affective Disorders Reports 4 (2021): 100094, 10.1016/j.jadr.2021.100094. PMC8623847 34841384

[51] S. Ashaba , C. E. Cooper‐Vince , D. Vorechovská , et al., “Community Beliefs, HIV Stigma, and Depression Among Adolescents Living With HIV in Rural Uganda,” African Journal of AIDS Research 18 (2019): 169–180, 10.2989/16085906.2019.1637912. PMC6774805 31339461

[52] L. Cluver , M. Orkin , M. E. Boyes , L. Sherr , D. Makasi , and J. Nikelo , “Pathways From Parental AIDS to Child Psychological, Educational and Sexual Risk: Developing an Empirically‐Based Interactive Theoretical Model,” Social Science & Medicine 87 (2013): 185–193, 10.1016/j.socscimed.2013.03.028. 23631794

[53] T. Sudjaritruk , L. Aurpibul , W. N. Songtaweesin , et al., “Integration of Mental Health Services Into HIV Healthcare Facilities Among Thai Adolescents and Young Adults Living With HIV,” Journal of the International AIDS Society 24 (2021): e25668, 10.1002/jia2.25668. PMC7876472 33569878

[54] S. Zietz , B. J. Iritani , F. A. Otieno , et al., “Suicide Behaviour Among Adolescents in a High HIV Prevalence Region of Western Kenya: A Mixed‐Methods Study,” Global Public Health 16 (2021): 88–102, 10.1080/17441692.2020.1782964. PMC7752827 32567992

[55] M. O. Folayan , O. Oginni , O. I. Ibigbami , et al., “Associations Between Mental Health and HIV Status Among Sexual Minority and Heterosexual Adolescents in Nigeria,” BMJ Global Health 7 (2022): e010231, 10.1136/bmjgh-2022-010231. PMC9791420 36564086

[56] K. Steventon Roberts , C. Smith , and E. Toska , “Risk Factors for Poor Mental Health Among Adolescent Mothers in South Africa,” Psychology, Health and Medicine 27 (2022): 67–84, 10.1080/13548506.2022.2124295. 36154770

[57] K. J. Steventon Roberts , C. Smith , and L. Cluver , “Adolescent Mothers and Their Children Affected by HIV—An Exploration of Maternal Mental Health, and Child Cognitive Development,” PLoS ONE 17 (2022): e0275805, 10.1371/journal.pone.0275805. PMC9584392 36264898

[58] K. J. Roberts , C. Smith , L. Cluver , et al., “Adolescent Motherhood and HIV in South Africa: Examining Prevalence of Common Mental Disorder,” AIDS and Behavior 26 (2022): 1197–1210, 10.1007/s10461-021-03474-8. PMC8940800 34570313

[59] S. Ashaba , C. Cooper‐Vince , D. Vorechovská , et al., “Development and Validation of a 20‐Item Screening Scale to Detect Major Depressive Disorder Among Adolescents With HIV in Rural Uganda: A Mixed‐Methods Study,” SSM—Population Health 7 (2019): 100332, 10.1016/j.ssmph.2018.100332. PMC6289958 30560198

[60] J. D. Namuli , J. S. Nalugya , P. Bangirana , and E. Nakimuli‐Mpungu , “Prevalence and Factors Associated With Suicidal Ideation Among Children and Adolescents Attending a Pediatric HIV Clinic in Uganda,” Frontiers in Sociology 6 (2021): 656739, 10.3389/fsoc.2021.656739. PMC8239397 34212027

[61] S. Adeyemo , O. G. Olorunkoya , O. L. Chinelo , et al., “Prevalence and Psychosocial Correlates of Suicidal Ideation Among Adolescents Living With HIV in Southwestern Nigeria, West Africa,” HIV and AIDS Review 18 (2019): 273–278, 10.5114/hivar.2019.85949.

[62] I. Ouansafi , D. Chibanda , E. Munetsi , and V. Simms , “Impact of Friendship Bench Problem‐Solving Therapy on Adherence to ART in Young People Living With HIV in Zimbabwe: A Qualitative Study,” PLoS ONE 16 (2021): e0250074, 10.1371/journal.pone.0250074. PMC8061927 33886619

[63] S. R. Taquette , O. de Rodrigues A , and L. R. Bortolotti , “Perception of Pre‐ and Post‐HIV Test Counseling Among Patients Diagnosed With Aids in Adolescence HIV Test Counseling for Adolescents,” Ciencia e Saude Coletiva 22 (2017): 23–30, 10.1590/1413-81232017221.23532015. 28076526

[64] B. D. Kennard , L. T. Brown , L. Hawkins , et al., “Development and Implementation of Health and Wellness CBT for Individuals With Depression and HIV,” Cognitive and Behavioral Practice 21 (2014): 237–246, 10.1016/j.cbpra.2013.07.003. PMC4002170 24795524

[65] L. Asadi , S. Esmaelzadeh , H. E. Koochak , A. Zareiyan , B. Khorsandi , and Z. Behboodi‐Moghadam , “Reproductive and Sexual Health Concerns in HIV‐Positive Youth, Aged 15 to 24 Years,” HIV & AIDS Review 22 (2023): 168–176, 10.5114/hivar.2023.127710.

[66] M. Lee , F. Eyer , N. Felgenhauer , H. H. Klinker , and C. D. Spinner , “Overdose of Dolutegravir in Combination With Tenofovir Disaproxil Fumarate/Emtricitabine in Suicide Attempt in a 21‐Year Old Patient,” AIDS Research and Therapy 12 (2015): 18, 10.1186/s12981-015-0054-y. PMC4455705 26045712

[67] T. Betancourt , M. C. Smith Fawzi , and A. Stevenson , “Ethics in Community‐Based Research With Vulnerable Children: Perspectives From Rwanda,” PLoS ONE 11 (2016): e0157042, 10.1371/journal.pone.0157042. PMC4924793 27351337

[68] B. S. Nsibandze , C. Downing , M. Poggenpoel , and C. P. Myburgh , “Experiences of Grandmothers Caring for Female Adolescents Living With HIV in Rural Manzini, Eswatini: A Caregiver Stress Model Perspective,” African Journal of AIDS Research 19 (2020): 123–134, 10.2989/16085906.2020.1758735. 32780678

[69] B. S. Nsibandze , C. Downing , M. Poggenpoel , and C. P. H. Myburgh , ““I Have Been Rejected So Many Times” Experiences of Female Adolescents Living With HIV in Rural Manzini, Eswatini: A Case Study,” International Journal of Africa Nursing Sciences 14 (2021): 100307, 10.1016/j.ijans.2021.100307.

[70] C. Jimu , K. Govender , R. Kanyemba , et al., “Experiences of Intimate Relationships, Stigma, Social Support and Treatment Adherence Among HIV‐Positive Adolescents in Chiredzi District, Zimbabwe,” African Journal of AIDS Research 20 (2021): 214–223, 10.2989/16085906.2021.1979059. 34635020

[71] B. L. Knizek , J. Mugisha , J. Osafo , et al., “Growing Up HIV‐Positive in Uganda: ‘Psychological Immunodeficiency’? A Qualitative Study,” BMC Psychology 30 (2017): 1–10, 10.1186/s40359-017-0199-7. PMC6389182 28841921

[72] M. Mutumba , V. Musiime , A. C. Tsai , et al., “Disclosure of HIV Status to Perinatally Infected Adolescents in Urban Uganda: A Qualitative Study on Timing, Process, and Outcomes,” Journal of the Association of Nurses in AIDS Care 26 (2015): 472–484, 10.1016/j.jana.2015.02.001. 26066697

[73] N. Willis , W. Mavhu , C. Wogrin , A. Mutsinze , and A. Kagee , “Understanding the Experience and Manifestation of Depression in Adolescents Living With HIV in Harare, Zimbabwe,” PLoS ONE 13 (2018): e0190423, 10.1371/journal.pone.0190423. PMC5752002 29298326

[74] E. Yang , S. Mphele , N. Moshashane , et al., “Distinctive Barriers to Antiretroviral Therapy Adherence Among Non‐Adherent Adolescents Living With HIV in Botswana,” AIDS Care 30 (2018): 224–231, 10.1080/09540121.2017.1344767. PMC6083824 28643572

[75] O. Yator , L. Khasakhala , G. J. Stewart , et al., “Acceptability and Impact of Group Interpersonal Therapy (IPT‐G) on Kenyan Adolescent Mothers Living With Human Immunodeficiency Virus (HIV): A Qualitative Analysis,” BMC Women's Health 22 (2022): 240, 10.1186/s12905-022-01807-w. PMC9206094 35717156

[76] R. Chhabra , N. Teitelman , E. J. Silver , J. Raufman , and L. J. Bauman , “Vulnerability Multiplied: Health Needs Assessment of 13–18‐Year‐Old Female Orphan and Vulnerable Children in Kenya,” World Medical & Health Policy 10 (2018): 129–145, 10.1002/wmh3.267.

[77] T. Biswas , J. G. Scott , K. Munir , et al., “Global Variation in the Prevalence of Suicidal Ideation, Anxiety and Their Correlates Among Adolescents: A Population Based Study of 82 Countries,” EClinicalMedicine 24 (2020): 100395, 10.1016/j.eclinm.2020.100395. PMC7525128 33015595

[78] A. R. Van Meter , E. A. Knowles , and E. H. Mintz , “Systematic Review and Meta‐Analysis: International Prevalence of Suicidal Ideation and Attempt in Youth,” Journal of the American Academy of Child and Adolescent Psychiatry 62 (2023): 973–986. 10.1016/j.jaac.2022.07.867 PMC12702477 36563876

[79] J. Piao , Y. Huang , C. Han , et al., “Alarming Changes in the Global Burden of Mental Disorders in Children and Adolescents From 1990 to 2019: A Systematic Analysis for the Global Burden of Disease Study,” European Child & Adolescent Psychiatry 31 (2022): 1827–1845, 10.1007/s00787-022-02040-4. 35831670

[80] K. L. Lovero , P. F. Dos Santos , A. X. Come , M. L. Wainberg , and M. A. Oquendo , “Suicide in Global Mental Health,” Current Psychiatry Reports 25 (2023): 255–262, 10.1007/s11920-023-01423-x. PMC10182355 37178317

[81] P. Bertuccio , A. Amerio , E. Grande , et al., “Global Trends in Youth Suicide From 1990 to 2020: An Analysis of Data From the WHO Mortality Database,” eClinicalMedicine 70 (2024): 102506, 10.1016/j.eclinm.2024.102506. PMC10911948 38440131

[82] C. R. Glenn , E. M. Kleiman , J. Kellerman , et al., “Annual Research Review: A Meta‐Analytic Review of Worldwide Suicide Rates in Adolescents,” Journal of Child Psychology and Psychiatry 61 (2020): 294–308, 10.1111/jcpp.13106. 31373003

[83] Z. K. Abraham and L. Sher , “Adolescent Suicide as a Global Public Health Issue,” International Journal of Adolescent Medicine and Health 31 (2017): 20170036, 10.1515/ijamh-2017-0036. 28686572

[84] J. Renaud , S. L. MacNeil , L. Vijayakumar , et al., “Suicidal Ideation and Behavior in Youth in Low‐ and Middle‐Income Countries: A Brief Review of Risk Factors and Implications for Prevention,” Frontiers in Psychiatry 13 (2022): 1044354, 10.3389/fpsyt.2022.1044354. PMC9763724 36561636

[85] P. R. Ward , R. Puspitasari , A. Rose , B. S. Gebremariyam , and N. K. Fauk , “Understanding HIV‐Related Mental Health Challenges and Contributing Factors Among Indonesian Adolescents Living With HIV,” International Journal of Environmental Research and Public Health 22 (2025): 83, 10.3390/ijerph22010083. PMC11764498 39857536

[86] J. R. MacLean and K. Wetherall , “The Association Between HIV‐Stigma and Depressive Symptoms Among People Living With HIV/AIDS: A Systematic Review of Studies Conducted in South Africa,” Journal of Affective Disorders 287 (2021): 125–137, 10.1016/j.jad.2021.03.027. 33780828

[87] J. W. Luk , R. B. Goldstein , J. Yu , D. L. Haynie , and S. E. Gilman , “Sexual Minority Status and Age of Onset of Adolescent Suicide Ideation and Behavior,” Pediatrics 148 (2021): e2020034900, 10.1542/peds.2020-034900. PMC9446478 34580171

[88] J. N. Fish , C. E. Rice , S. T. Lanza , et al., “Is Young Adulthood a Critical Period for Suicidal Behavior Among Sexual Minorities? Results From a US National Sample,” Prevention Science 20 (2019): 353–365. 10.1007/s11121-018-0878-5 PMC6163093 29594980

[89] K. R. Agarwal , S. A. Fleary , P. Kreniske , and C. A. Teasdale , “Bullying and Suicide Attempts Among US High School Students,” JAMA Network Open 9 (2026): e2552089, 10.1001/jamanetworkopen.2025.52089. PMC12761328 41481296

[90] A. J. Lyons , S. A. Fleary , P. Kreniske , and C. A. Teasdale , “Parental Emotional Abuse, Sexual Identity, and Adolescent Suicide Attempts During the COVID‐19 Pandemic,” The Journal of Adolescent Health: Official Publication of the Society for Adolescent Medicine 76, no. 3 (2025): 401–407, https://doi.org.sacredheart.idm.oclc.org/10.1016/j.jadohealth.2024.10.029. 10.1016/j.jadohealth.2024.10.029 39674946

[91] B. Armoon , M.‐J. Fleury , A.‐H. Bayat , et al., “HIV Related Stigma Associated With Social Support, Alcohol Use Disorders, Depression, Anxiety, and Suicidal Ideation Among People Living With HIV: A Systematic Review and Meta‐Analysis,” International Journal of Mental Health Systems 16 (2022): 17, 10.1186/s13033-022-00527-w. PMC8896327 35246211

[92] R. E. Gearing and D. Alonzo , “Religion and Suicide: New Findings,” Journal of Religion and Health 57 (2018): 2478–2499, 10.1007/s10943-018-0629-8. 29736876

[93] S. C. Campisi , B. Carducci , N. Akseer , C. Zasowski , P. Szatmari , and Z. A. Bhutta , “Suicidal Behaviours Among Adolescents From 90 Countries: A Pooled Analysis of the Global School‐Based Student Health Survey,” BMC Public Health [Electronic Resource] 20 (2020): 1102, 10.1186/s12889-020-09209-z. PMC7416394 32772922

[94] T. C. Seunanden , N. Ngwenya , and J. Seeley , “Experiences and Perceptions on Antiretroviral Therapy Adherence and Non‐Adherence: A Scoping Review of Young People Living With HIV in Sub‐Saharan Africa,” BMC Public Health [Electronic Resource] 25 (2025): 1450, 10.1186/s12889-025-22579-6. PMC12004696 40247282

[95] Y. Kiirya , S. Kitaka , J. Kalyango , et al., “Acceptability of an Online Peer Support Group as a Strategy to Improve Antiretroviral Therapy Adherence Among Young People in Kampala District, Uganda: Qualitative Findings,” BMC Infectious Diseases 25 (2025): 461, 10.1186/s12879-025-10831-8. PMC11969772 40181265

[96] C. Mugo , D. Seeh , B. Guthrie , et al., “Association of Experienced and Internalized Stigma With Self‐Disclosure of HIV Status by Youth Living With HIV,” AIDS and Behavior 25 (2021): 2084–2093, 10.1007/s10461-020-03137-0. PMC8768004 33389374

[97] H. Hjelmeland and B. L. Knizek , “Time to Change Direction in Suicide Research,” in The International Handbook of Suicide Prevention: Second Edition, ed. C. Rory and J. P. O'Connor (2016), 696–709, 10.1002/9781118903223.

[98] P. Kreniske , H. Isenberg , F. Nalugoda , et al., “Improving Understanding of Capacity to Consent to Sensitive HIV Biomedical Prevention Research Among Adolescents and Their Parents or Guardians in Rakai Uganda (ICARE): A Quasi‐Experimental Nonequivalent Groups Design Protocol,” BMC Medical Ethics 27 (2025): 2, 10.1186/s12910-025-01342-x. PMC12781717 41327175

[99] A. Knopf , C. B. Draucker , and J. Dennis Fortenberry , “Parental Engagement in Consent Processes for Enrollment in Biomedical HIV Prevention Trials: Implications for Minor Adolescents' Willingness to Participate,” Journal of Adolescent Health 72 (2023): 703–711, 10.1016/j.jadohealth.2022.11.241. PMC11114099 36646563

[100] B. Mathews , “Adolescent Capacity to Consent to Participate in Research: A Review and Analysis Informed by Law, Human Rights, Ethics, and Developmental Science,” Laws 12 (2023): 2.

[101] S. Day , B. G. Kapogiannis , S. K. Shah , et al., “Adolescent Participation in HIV Research: Consortium Experience in Low and Middle‐Income Countries and Scoping Review,” Lancet HIV 7 (2020): e844–e852, 10.1016/S2352-3018(20)30269-1. PMC8491773 33275917

[102] P. Kreniske , S. Hoffman , W. Ddaaki , et al., “Capacity to Consent to Research Among Adolescent−Parent Dyads in Rakai, Uganda,” Journal of Pediatrics 257 (2023): 113271, 10.1016/j.jpeds.2022.11.012. PMC10202026 36402433

[103] G. Parmigiani , M. Benevento , B. Solarino , et al., “Decisional Capacity to Consent to Treatment in Children and Adolescents: A Systematic Review,” Psychiatry Research 344 (2025): 116343, 10.1016/j.psychres.2024.116343. 39798483

[104] “Confidential Healthcare for Adolescent Minors and Young Adults: A Position Paper of the Society for Adolescent Health and Medicine,” Journal of Adolescent Health 77 (2025): 791–796, 10.1016/j.jadohealth.2025.06.006. 40973393

[105] A. Bhana , P. Kreniske , A. Pather , M. A. Abas , and C. A. Mellins , “Interventions to Address the Mental Health of Adolescents and Young Adults Living With or Affected by HIV: State of the Evidence,” Journal of the International AIDS Society 24, no. 2 (2021): e25713, 10.1002/JIA2.25713. PMC8222850 34164939

[106] H. M. Richards and L. J. Schwartz , “Ethics of Qualitative Research: Are There Special Issues for Health Services Research?,” Family Practice 19, no. 2 (2002): 135–139, 10.1093/fampra/19.2.135. 11906977

[107] S. A. Safren , C. M. O'Cleirigh , J. R. Bullis , M. W. Otto , M. D. Stein , and M. H. Pollack , “Cognitive Behavioral Therapy for Adherence and Depression (CBT‐AD) in HIV‐Infected Injection Drug Users: A Randomized Controlled Trial,” Journal of Consulting and Clinical Psychology 80, no. 3 (2012): 404–415, 10.1037/a0028208. PMC3365619 22545737

[108] J. J. Mootz , C. Chantre , K. Sikkema , et al., “Leveraging a Digitized Mental Wellness (DIGImw) Program to Provide Mental Health Care for Internally Displaced People,” Psychiatric Services 75, no. 1 (2024): 98–101, 10.1176/appi.ps.202100552. PMC10794516 37461818

[109] K. Källander , J. K. Tibenderana , O. J. Akpogheneta , et al., “Mobile Health (mHealth) Approaches and Lessons for Increased Performance and Retention of Community Health Workers in Low‐ and Middle‐Income Countries: A Review,” Journal of Medical Internet Research 15 (2013): e17, 10.2196/jmir.2130. PMC3636306 23353680

[110] P. Kreniske , O. I. Namuyaba , R. Kasumba , et al., “Mobile Phone Technology for Preventing HIV and Related Youth Health Problems, Sexual Health, Mental Health, and Substance Use Problems in Southwest Uganda (Youth Health SMS): Protocol for a Pilot Randomized Controlled Trial,” JMIR Research Protocols 12 (2023): e49352, 10.2196/49352. PMC10762611 38113102

[111] M. Brooks , B. Burmen , A. Olashore , et al., “Symptoms of Depression, Anxiety, and Thoughts of Suicide/Self‐Injury in Adolescents and Young Adults Living With HIV in Botswana,” African Journal of AIDS Research 22 (2023): 54–62, 10.2989/16085906.2023.2186252. PMC10787227 37116112

[112] M. C. S. Fawzi , L. Ng , F. Kanyanganzi , et al., “Mental Health and Antiretroviral Adherence Among Youth Living With HIV in Rwanda,” Pediatrics 138 (2016): e20153235, 10.1542/peds.2015-3235. PMC5051202 27677570

[113] D. Gaitho , M. Kumar , D. Wamalwa , G. N. Wambua , and R. Nduati , “Understanding Mental Health Difficulties and Associated Psychosocial Outcomes in Adolescents in the HIV Clinic at Kenyatta National Hospital, Kenya,” Annals of General Psychiatry 29 (2018): 1–9, 10.1186/s12991-018-0200-8. PMC6038280 30002721

[114] E. Kemigisha , B. Zanoni , K. Bruce , et al., “Prevalence of Depressive Symptoms and Associated Factors Among Adolescents Living With HIV/AIDS in South Western Uganda,” AIDS Care 31 (2019): 1297–1303, 10.1080/09540121.2019.1566511. PMC6614012 30621430

[115] M. H. Kim , A. C. Mazenga , A. Devandra , et al., “Prevalence of Depression and Validation of the Beck Depression Inventory‐II and the Children's Depression Inventory‐Short Amongst HIV‐Positive Adolescents in Malawi,” Journal of the International AIDS Society 17 (2014): 18965, 10.7448/IAS.17.1.18965. PMC4119288 25085002

[116] L. Sherr , A. R. Yakubovich , S. Skeen , et al., “How Effective Is Help on the Doorstep? A Longitudinal Evaluation of Community‐Based Organisation Support,” PLoS ONE 11 (2016): e0151305, 10.1371/journal.pone.0151305. PMC4788449 26967732

[117] L. Sherr , L. D. Cluver , E. Toska , et al., “Differing Psychological Vulnerabilities Among Behaviourally and Perinatally HIV Infected Adolescents in South Africa–Implications for Targeted Health Service Provision,” AIDS Care 30, no. sup2 (2018): 92–101, 10.1080/09540121.2018.1476664. 29848010

