# Supplementary material for: Understanding Suicidality Among Young People Living With and Affected by HIV: A Systematic Review
Source: J Int AIDS Soc. 2026 Nov 9;29(11):e70193. doi: 10.1002/jia2.70193 (PMC13748812; doi:10.1002/jia2.70193)
Supplement: Supplementary file 1 [file JIA2-29-e70193-s001.docx]

**Appendix A: Systematic Search Strategy**

The following databases were searched:

- PubMed
- Cochrane
- Scopus
- Joanna Briggs Institute (JBI)

The search strategy was to combine searches of:

- “HIV” and “suicide” and “adolescent” related terms
- “HIV” and “mental disorder” and “adolescent” related terms

**HIV Terms:**

1. HIV
2. Acquired immunodeficiency syndrome
3. Human immunodeficiency virus
4. AIDS
5. Acquired immunodeficiency syndrome

**Suicide Terms:**

1. Suicide
2. Attempted suicide
3. Suicidal ideation
4. Suicide completion
5. Suicide attempt
6. Completed suicide

**Mental Disorder Terms:**

1. Mental disorders
2. Mental health
3. Depressive disorder
4. Depressive disorders

**Age Terms:**

1. Adolescent
2. Young adult
3. Minors
4. School student
5. Boy
6. Girl
7. NEETs
8. NEET
9. Pediatric
10. Paediatric
11. School age
12. Preteen
13. Preadolescen
14. Youngster
15. Juvenile
16. School-age youth
17. Childhood
18. Teenager
19. Kid

**Databases were also searched for specific types of reviews using the following search terms:**

| **Database**  **(Search Date)** | **Search Terms** |
| --- | --- |
| PubMed  (August 14^th^, 2023) | ("HIV"[Mesh] OR "Acquired Immunodeficiency Syndrome"[Mesh] OR "human immunodeficiency virus"[tiab] OR “AIDS”[tiab] OR "acquired immunodeficiency syndrome"[tiab]) AND ("Suicide"[Mesh] OR Suicide[tiab] OR “Attempted Suicide"[tiab] OR “Suicidal Ideation”[tiab] OR “Suicide Completion”[tiab] OR “Suicide Attempt"[tiab] OR “Completed Suicide"[tiab]) AND (“Adolescent”[Mesh] OR “Young Adult”[Mesh] OR “Minors”[tiab] OR “School Student”[tiab] OR “Boy”[tiab] OR “Girl”[tiab] OR “NEETs”[tiab] OR “NEET”[tiab] OR “Pediatric”[tiab] OR “Paediatric”[tiab] OR “School Age”[tiab] OR “Preteen”[tiab] OR “Preadolescen”[tiab] OR “Youngster”[tiab] OR “School-Age Youth”[tiab] OR “Childhood”[tiab] OR “Teenager”[tiab] OR “Kid”[tiab])  (“HIV"[Mesh] OR "Acquired Immunodeficiency Syndrome"[Mesh] OR "human immunodeficiency virus"[tiab] OR “AIDS”[tiab] OR "acquired immunodeficiency syndrome"[tiab]) AND ("Mental Disorders"[Mesh] OR “Mental Health”[tiab]) AND (“Adolescent”[Mesh] OR “Young Adult”[Mesh] OR “Minors”[tiab] OR “School Student”[tiab] OR “Boy”[tiab] OR “Girl”[tiab] OR “NEETs”[tiab] OR “NEET”[tiab] OR “Pediatric”[tiab] OR “Paediatric”[tiab] OR “School Age”[tiab] OR “Preteen”[tiab] OR “Youngster”[tiab] OR “School-Age Youth”[tiab] OR “Childhood”[tiab] OR “Teenager”[tiab] OR “Kid”[tiab]) |
| Cochrane  (August 14^th^, 2023) | (HIV or Acquired Immunodeficiency Syndrome or human immunodeficiency virus* or AIDS* or acquired immunodeficiency syndrome*) NEAR/10 (Suicide* or Attempted Suicide* or Suicide Completion* or Suicide Attempt* or Suicidal Ideation*) NEAR/10 (Adolescent* or Young Adult* or Minors*or Boy* or Girl* or NEETs* or NEET* Pediatric* or Paediatric* or School Age* or Preteen* or Preadolescen* or Youngster* or Juvenile* or School-Age Youth* or Childhood* or Teenager* or Kid*)  (HIV or Acquired Immunodeficiency Syndrome or human immunodeficiency virus* or AIDS* or acquired immunodeficiency syndrome*) NEAR/10 (Mental Disorder* or Depressive Disorder*) NEAR/10 (Adolescent* or Young Adult* or Minors* or Boy* or Girl* or NEETs* or NEET* Pediatric* or Paediatric* or School Age* or Preteen* or Preadolescen* or Youngster* or Juvenile* or School-Age Youth* or Childhood*or Teenager* or Kid*) |
| Scopus  (August 14^th^, 2023) | ("HIV" OR "Acquired Immunodeficiency Syndrome" OR “human immunodeficiency virus” OR “AIDS” OR “acquired immunodeficiency syndrome”) AND ("Suicide" OR "Suicide, Attempted" OR "Suicide, Completed" OR "Suicidal Ideation" OR "Attempted Suicide" OR "Suicide Completion" OR "Suicide Attempt" OR "Completed Suicide") AND (“Adolescent” OR “Young Adult” OR “Minors” OR “School Student” OR “Boy” OR “Girl” OR “NEETs” OR “NEET” OR “Pediatric” OR “Paediatric” OR “School Age” OR “Preteen” OR “Preadolescen” OR “Youngster” OR “Juvenile” OR “School-Age Youth” OR “Childhood” OR “Teenager” OR “Kid”)  ("HIV" OR "Acquired Immunodeficiency Syndrome" OR “human immunodeficiency virus” OR “AIDS” OR “acquired immunodeficiency syndrome”) AND ("Mental Disorder" OR "Depressive Disorders") AND (“Adolescent” OR “Young Adult” OR “Minors” OR “School Student” OR “Boy” OR “Girl” OR “NEETs” OR “NEET” OR “Pediatric” OR “Paediatric” OR “School Age” OR “Preteen” OR “Preadolescen” OR “Youngster” OR “Juvenile” OR “School-Age Youth” OR “Childhood” OR “Teenager” OR “Kid”) |
| JBI  (October 5^th^, 2023) | (HIV or Acquired Immunodeficiency Syndrome or human immunodeficiency virus* or AIDS* or acquired immunodeficiency syndrome*) AND (Suicide* or Attempted Suicide* or Suicide Completion* or Suicide Attempt* or Suicidal Ideation*) AND (Adolescent* or Young Adult* or Minors*or Boy* or Girl* or NEETs* or NEET* Pediatric* or Paediatric* or School Age* or Preteen* or Preadolescen* or Youngster* or Juvenile* or School-Age Youth* or Childhood* or Teenager* or Kid*)  (HIV or Acquired Immunodeficiency Syndrome or human immunodeficiency virus* or AIDS* or acquired immunodeficiency syndrome*) AND (Mental Disorder* or Depressive Disorder*) AND (Adolescent* or Young Adult* or Minors* or Boy* or Girl* or NEETs* or NEET* Pediatric* or Paediatric* or School Age* or Preteen* or Preadolescen* or Youngster* or Juvenile* or School-Age Youth* or Childhood*or Teenager* or Kid*) |

**Appendix B. Detailed MMAT Ratings for All Included Studies**

| Study Type  Author, publication year | Quality Criterion 1 | Quality Criterion 2 | Quality Criterion 3 | Quality Criterion 4 | Quality Criterion 5 | Total Score | % |
| --- | --- | --- | --- | --- | --- | --- | --- |
| **Mixed Methods Studies** | **Is there an adequate rationale for using a mixed methods design to address the research question?** | **Are the different components of the study effectively integrated to answer the research question?** | **Are the outputs of the integration of qualitative and quantitative components adequately interpreted?** | **Are divergences and inconsistencies between quantitative and qualitative results adequately addressed?** | **Do the different components of the study adhere to the quality criteria of each tradition of the methods involved?** |  |  |
| Ashaba 2019[59] | 1 | 1 | 0.5 | 0.5 | 0.5 | 3.5 | 70 |
| Betancourt 2016[67] | 0 | 1 | 0.5 | 0.5 | 1 | 3 | 60 |
| Chhabra 2018[76] | 1 | 1 | 0.5 | 0.5 | 0.5 | 3.5 | 70 |
| Kennard 2014[64] | 0 | 1 | 1 | 0.5 | 0.5 | 3 | 60 |
| Zietz 2021[54] | 1 | 1 | 1 | 1 | 0.5 | 4.5 | 90 |
| **Qualitative Studies** | **Is the qualitative approach appropriate to answer the research question?** | **Are the qualitative data collection methods adequate to address the research question?** | **Are the findings adequately derived from the data?** | **Is the interpretation of results sufficiently substantiated by data?** | **Is there coherence between qualitative data sources, collection, analysis and interpretation?** |  |  |
| Asadi 2023[65] | 1 | 1 | 0.5 | 0.5 | 0.5 | 3.5 | 70 |
| Ashaba 2019[51] | 1 | 1 | **1** | **1** | **1** | 5 | 100 |
| Jimu 2021[70] | 1 | 1 | 1 | 1 | 1 | 5 | 100 |
| Kip 2022[9] | 1 | 1 | 0.5 | 0.5 | 1 | 4 | 80 |
| Knizek 2017[71] | 1 | 1 | 0.5 | 0.5 | 0.5 | 3.5 | 70 |
| Mutumba 2015[72] | 1 | 1 | 1 | 1 | 1 | 5 | 100 |
| Nsibandze 2020[68] | 1 | 1 | 1 | 1 | 1 | 5 | 100 |
| Nsibandze 2021[69] | 1 | 0.5 | 1 | **1** | 1 | 4.5 | 90 |
| Ouansafi 2021[62] | 1 | 1 | 1 | 1 | 1 | 5 | 100 |
| Taquette 2017[63] | 1 | 1 | 0.5 | 1 | **1** | 4.5 | 90 |
| Willis 2018[73] | 1 | 1 | 1 | 1 | 1 | 5 | 100 |
| Yang 2018[74] | 1 | 1 | 1 | 1 | 1 | 5 | 100 |
| Yator 2022[75] | 0.5 | 1 | 1 | 1 | **1** | 4.5 | 90 |
| **Quantitative Studies** | **Is the sampling strategy relevant to address the research question?** | **Is the sample representative of the target population?** | **Are the measurements appropriate?** | **Is the risk of nonresponse bias low?** | **Is the statistical analysis appropriate to answer the research question?** |  |  |
| Adeyemo 2019[61] | 0.5 | 0.5 | 1 | 0.5 | 1 | 3.5 | 70 |
| Arnold 2023[39] | 0.5 | 0.5 | 1 | 1 | 1 | 4 | 80 |
| Ashaba 2021[50] | 1 | 0.5 | 1 | 0.5 | 1 | 4 | 80 |
| Aurpibul 2023[36] | 1 | 0.5 | 1 | 1 | 1 | 4.5 | 90 |
| Brittain 2019[42] | 0.5 | 0.5 | 1 | 0.5 | 1 | 3.5 | 70 |
| Brooks 2023[111] | 0.5 | 0.5 | 1 | 1 | 1 | 4 | 80 |
| Casale 2019[43] | 1 | 1 | 1 | 0.5 | 1 | 4.5 | 90 |
| Cluver 2013[52] | 1 | 1 | 1 | 0.5 | 1 | 4.5 | 90 |
| Fawzi 2016[112] | 0.5 | 0.5 | 1 | 0.5 | 1 | 3.5 | 70 |
| Folayan 2022[55] | 1 | 0.5 | 1 | 0.5 | 1 | 4 | 80 |
| Gaitho 2018[113] | 1 | 0.5 | 1 | 0.5 | 1 | 4 | 80 |
| Gross 2016[31] | 1 | 0.5 | 1 | 1 | 1 | 4.5 | 90 |
| Haas 2020[49] | 1 | 0.5 | 0.5 | 0.5 | 1 | 3.5 | 70 |
| Kemigisha 2019[114] | 0.5 | 0.5 | 1 | 0.5 | 1 | 3.5 | 70 |
| Kim 2014[115] | 1 | 0.5 | 1 | 0.5 | 1 | 4 | 80 |
| Kipke 2020[32] | 1 | 0 | 1 | 0.5 | 1 | 3.5 | 70 |
| Kreniske 2019[46] | 1 | 0.5 | 1 | 0.5 | 1 | 4 | 80 |
| Kreniske 2021[40] | 1 | 0.5 | 1 | 0.5 | 1 | 4 | 80 |
| Liotta 2023[33] | 1 | 0.5 | 1 | 0.5 | 1 | 4 | 80 |
| Mirani 2015[47] | 1 | 0.5 | 0.5 | 0.5 | 1 | 3.5 | 70 |
| Namuli 2021[60] | 1 | 0.5 | 1 | 1 | 1 | 4.5 | 90 |
| Ndongo 2023[38] | 0.5 | 0.5 | 1 | **1** | 1 | 4 | 80 |
| Ng 2015[41] | 1 | 1 | 1 | 0.5 | 1 | 4.5 | 90 |
| Orrigio 2021[48] | 1 | **1** | 0.5 | 0.5 | 1 | 4 | 80 |
| Roberts 2022[58] | 1 | 1 | 1 | 1 | 1 | 5 | 100 |
| Rukundo 2020[44] | 0.5 | 0.5 | 1 | 0.5 | 1 | 3.5 | 70 |
| Sherr 2016[116] | 1 | 0.5 | 1 | 0.5 | 1 | 4 | 80 |
| Sherr 2018[117] | 1 | 0.5 | 1 | 1 | 1 | 4.5 | 90 |
| Sohn 2023[35] | 1 | 0.5 | 1 | 0.5 | 1 | 4 | 80 |
| Steventon Roberts 2022[57] | 1 | 0.5 | 1 | 0.5 | 1 | 4 | 80 |
| Steventon Roberts 2022[56] | 1 | 1 | 1 | 0 | 1 | 4 | 80 |
| Sudjaritruk 2021[53] | 1 | 0.5 | 1 | 1 | 1 | 4.5 | 90 |
| Walsh 2017[34] | 1 | 0.5 | 1 | 0 | 1 | 3.5 | 70 |
| Wonde 2019[37] | 1 | 1 | 1 | 0.5 | 1 | 4.5 | 90 |
| Woollett 2017[45] | 0.5 | 0.5 | 1 | 0.5 | 1 | 3.5 | 70 |

Scoring: “Yes” (1), “No” (0) or “Can’t tell” (0.5)
